# Dystrophin deficiency leads to dysfunctional glutamate clearance in iPSC derived astrocytes

**DOI:** 10.1038/s41398-019-0535-1

**Published:** 2019-08-21

**Authors:** Abdulsamie M. Patel, Keimpe Wierda, Lieven Thorrez, Maaike van Putten, Jonathan De Smedt, Luis Ribeiro, Tine Tricot, Madhavsai Gajjar, Robin Duelen, Philip Van Damme, Liesbeth De Waele, Nathalie Goemans, Christa Tanganyika-de Winter, Domiziana Costamagna, Annemieke Aartsma-Rus, Hermine van Duyvenvoorde, Maurilio Sampaolesi, Gunnar M. Buyse, Catherine M. Verfaillie

**Affiliations:** 1 Stem Cell Institute Leuven, Dept. of Development and Regeneration, KU Leuven, Leuven, Belgium; 2 Center for Brain & Disease Research, VIB, Leuven, Belgium; 3 KU Leuven Department of Development and Regeneration, Campus Kulak, Kortrijk, Belgium; 4 Department of Human Genetics, Leiden University Medical Center, Leiden, The Netherlands; 5 Translational Cardiomyology Lab, Stem Cell Biology and Embryology Unit, KU Leuven, Leuven, Belgium; 6 Laboratory of Neurobiology, Department of Neuroscience, KU Leuven, Leuven, Belgium; 7 Neurology Department, University Hospitals Leuven, Leuven, Belgium; 8 Department of Paediatric Child Neurology, University Hospitals Leuven, Leuven, Belgium; 9 Vesalius Research Center, Laboratory of Neurobiology, KU Leuven, Leuven, Belgium; 10 Laboratory for Diagnostic Genome Analysis, Leiden University Medical Center, Leiden, The Netherlands

**Keywords:** Stem cells, Autism spectrum disorders

## Abstract

Duchenne muscular dystrophy (DMD) results, beside muscle degeneration in cognitive defects. As neuronal function is supported by astrocytes, which express dystrophin, we hypothesized that loss of dystrophin from DMD astrocytes might contribute to these cognitive defects. We generated cortical neuronal and astrocytic progeny from induced pluripotent stem cells (PSC) from six DMD subjects carrying different mutations and several unaffected PSC lines. DMD astrocytes displayed cytoskeletal abnormalities, defects in Ca^+2^ homeostasis and nitric oxide signaling. In addition, defects in glutamate clearance were identified in DMD PSC-derived astrocytes; these deficits were related to a decreased neurite outgrowth and hyperexcitability of neurons derived from healthy PSC. Read-through molecule restored dystrophin expression in DMD PSC-derived astrocytes harboring a premature stop codon mutation, corrected the defective astrocyte glutamate clearance and prevented associated neurotoxicity. We propose a role for dystrophin deficiency in defective astroglial glutamate homeostasis which initiates defects in neuronal development.

## Introduction

Duchenne muscular dystrophy (DMD), the most common muscular dystrophy worldwide, is caused by mutations in the X-chromosome positioned *DMD* gene. DMD is characterized by progressive skeletal and cardiac muscle weakness, causing death in the second or third decade of life. In ± 30% of subjects, neurocognitive defects are also present^1^. The mean full-scale intelligence quotient of DMD subjects is ~1 standard deviation below normal^2^, and DMD subjects exhibit variable degrees of neurocognitive difficulties and display specific learning disorders, such as dyslexia^3,4^. In addition, a higher incidence of autism spectrum disorder (ASD), attention deficit hyperactivity disorder (ADHD) and obsessive-compulsive disorder (OCD) has been described^5^. Unlike the loss of muscle function, cognitive defects are not progressive and they do not correlate with the severity of the muscle disease^1^.

Given the critical role of dystrophin as an essential link between the dystrophin associated protein complex (DAPC) at the sarcolemma and the cytoskeleton, studies suggested that the loss of functional dystrophin in the brain may cause defective synapses^6^. Subsequent studies suggested a number of possible mechanisms that might underlay defective central nervous system (CNS) homeostasis in *mdx* mice, a mouse model for DMD^6,7^. Expression of dystrophin is controlled by three upstream promoters, which produce the full-length dystrophin isoform (Dp427) and four internal promoters that regulate production of shorter dystrophin isoforms (Dp260, Dp140, Dp116, and Dp71). In the CNS, the dystrophin isoforms Dp427c, Dp140, and Dp71 are expressed mostly in blood vessels and astrocytes^8,9^. While indirect evidence exists, linking the loss of different dystrophin isoforms with varying phenotypic outcomes such as, neuromuscular synaptic dysfunction^10^, decrease in whole brain volume^11,12^ and altered cerebral architecture^13,14^, all leading to cognitive issues^15^, no causal molecular and cellular basis have been described to date. Whereas the majority of post-mortem brain studies in autism have focused on uncovering neuronal abnormalities, recent evidence has demonstrated alterations in both microglial and astrocytic markers within the autistic brain^16–19^. In addition, the overlapping features between DMD-associated cognitive phenotypes and those reported for other syndromic cerebral pathologies^20–23^ and cognitive dysfunctions, including learning and memory, suggest that defects in DMD astrocytes may affect neuronal circuitry^24–28^.

Astrocytes are the most abundant glial cells in the central nervous system (CNS) and are not merely passive bystanders of nervous system development and maintenance. Besides being fundamental to neuronal nutrition via glycogen synthesis, storage and release, astrocytes also dictate the molecular homeostasis of the CNS by ion buffering, and neurotransmitter recycling (e.g. glutamate, γ-aminobutyric acid (GABA) and glycine)^29,30^. Astrocytes are morphologically complex, and tightly integrated into neural networks. Although neurogenesis precedes astrogenesis in the cortex, neuronal synapses only begin to form after astrocytes have been generated, concurrent with neuronal branching and process elaboration^17,31^. In the case of the main excitatory neurotransmitter in the mammalian CNS, Glutamate, its excess in the synaptic and extra-synaptic space leads to neuronal hyperexcitation and subsequent neuronal death, in a process known as “glutamate excitotoxicity”, which accompanies several inflammatory and neurodegenerative diseases of the CNS^16,32–34^. Therefore, unused glutamate during synaptic transmission must be rapidly cleared from the extracellular space. The mission of glutamate clearance is achieved, primarily, by astrocytes and is mediated by glutamate uptake transporters (EAAT-1 and EAAT-2 (known in rodents as GLAST and GLT-1))^29,30,35,36^. Of note is also the astrocytic release of trace amounts of glutamate to the adjacent neurons, which help to synchronize their firing and modulate their excitatory or inhibitory transmission^16,29,36,37^. Accordingly, by controlling the balance between glutamate uptake and release, glutamate homeostasis is achieved in CNS.

Overall, astrocytes are essentially brain “homeostatic cells” wherein, extracellular signals (inflammatory response, neurotrophic factors, neurotransmitters etc.) from astrocytes orchestrate a carefully balanced formation of neural circuits by powerfully controlling synapse formation, function, and elimination^30^. Alterations and loss of these critical astroglial functions have thus been hypothesized to contribute to most if not all cerebral pathologies. The modifications of astroglia in neuropathology are multifaceted, often disease-specific and may undergo a spectrum of differential metamorphoses during the course of pathological evolution^32,33^. As both synaptogenesis disruption and neuroinflammation are reported in autism, a role for astrocyte (and microglia downstream) involvement in the pathophysiology of autism has begun to receive an increasing amount of attention^21,38^.

Therefore, we hypothesized that dystrophin-deficient astrocytes may cause neuronal dysfunction that contribute to the neurocognitive or neuropsychiatric defects observed in DMD patients. To test this hypothesis, we created induced pluripotent stem cells (iPSCs) from six DMD subjects, from which we differentiated cortical as well as astrocytic progeny. DMD astrocytes displayed an abnormal activated-like morphology, defects in Ca^+2^ handling^39,40^, nitric oxide (NO) signaling^41,42^, and reactive oxygen species (ROS) accumulation^43^. Transcriptome analysis and functional studies demonstrated that DMD astrocytes display defective glutamate handling, including decreased removal and release of glutamate causing defective neurite outgrowth, which could be blocked by either ionotropic glutamate transporters/receptor blockers or *N*-methyl-d-aspartate (NMDA) receptor antagonists. Studies wherein iPSCs from a DMD subject with a premature stop codon mutation were treated with read-through molecules^44^, demonstrated not only partial restoration of dystrophin expression in iPSC-derived astrocytes, but also correction of the defective glutamate handling and associated neural toxicity.

## Materials and methods

### Study subjects

The protocol to obtain blood or fibroblasts for the generation of induced pluripotent stem cells from DMD subjects and normal donors was approved by the Ethics Committee of the University Hospitals Leuven (Clinical Trial Center (CTC), UZ Leuven, Campus Gasthuisberg #S55438, 31 Jul 2013). Consent was obtained from the DMD subjects’ guardian and an assent from the DMD subject himself to donate 5–10 cc of blood; while for D2, a skin biopsy was obtained as a source of fibroblasts for reprogramming. The blood samples as well as the skin biopsy were immediately transferred to Stem Cell Institute Leuven (SCIL) on ice. Subject information can be found in Supplementary Table 1. All cell lines utilized in this manuscript are summarized in Supplementary Table 2.

Materials, Primers, and antibodies used

Antibodies are listed in Supplementary table 3

Primer sequences are shown in Supplementary table 4

Catalogue numbers for materials used are listed in the Key Resource Table.

### Cell cultures

hESCs and human iPSCs were maintained on Geltrex LDEV-Free hESC-Qualified Reduced Growth Factor Basement Membrane Matrix (A1413302, Gibco *(TM)*) in Essential *(TM)* 8 medium (A1517001, Gibco *(TM)*) with 1000 U/ml penicillin–streptomycin. Colonies were routinely passaged with 0.5 mM EDTA (15575-020, Invitrogen) in Dulbecco’s phosphate-buffered saline (DPBS). Cultures were routinely analyzed by PCR for mycoplasma contamination.

### Derivation of induced pluripotent stem cells

For reprogramming with Sendai viral vectors, Cytotune 1 and Cytotune 2 non-integrating Sendai virus kits were purchased from Thermo Fisher, US and reprogramming was carried out per the kit’s instructions. In brief, peripheral blood mononuclear cells were isolated from five donors with yields varying between 0.43–4.5 × 10^6^/mL with 88–98 % cell viability^1^. For D2, cryopreserved fibroblasts were utilized for reprogramming. Fibroblasts were thawed at passage 2 and grown to 60% confluence before initiating the reprogramming protocol. Both the fibroblast and peripheral blood mononuclear cells were transduced at a density of 250,000 cells per well of a 6-well plate (Corning, USA) at a multiplicity of infection of 3. Approximately 1 week after transduction, the first morphological changes were observed. The first fully reprogrammed iPSC colonies were picked on D29, based on morphology. D1 and D2 were initially derived on mouse embryonic fibroblasts feeder layer, and subsequently adapted to a feeder-free culture system. Cells from D3 to D6 were directly derived as feeder-free iPSCs on hESC‐qualified matrigel (Becton Dickinson, BD, Belgium) and Essential 8 Medium (in a humidified incubator (5% CO2, 37 °C)). Once established and following quality control, all iPSCs were maintained in 6-well plates, fed with fresh media daily, and passaged every 7–8 days, and then frozen for further analysis.

### Generation of NSCs, cortical neurons, and astrocytes from hPSCs

To induce NSCs, PSCs were cultured to 100% confluence on BD matrigel in Essential 8 medium, then switched to a N2B27-based neural induction medium (NIM), which is Neural maintenance medium (NMM) supplemented with the small molecules SB431542 (10 μM, Stemgent, US) and Dorsomorphin (2 μM, Stemgent) for “dual-SMAD inhibition” for 14 days, based on previously described methods^3^. NMM consisted of 1:1 mix of DMEM/F12 and neurobasal media, supplemented with N2 (1:200), B27 (1:100), glutamax (1:100), NEAA (1:200), β-mercaptoethanol (1:2000), Pen/Strep (1:200) (all from ThermoFisher Scientific) and 2.5 µg/ml of insulin (Sigma Aldrich, Belgium). On day 12–14, neuro-epithelial like cells were detached using dispase (Sigma Aldrich) and replated on polyornithine-Laminin (both from ThermoFisher Scientific) coated surfaces. NMM was now supplemented with 20 ng/ml fibroblast growth factor (FGF2) (Peprotech, UK) and 5 µg/ml Heparin (Sigma Aldrich). NSCs were cultured in this expansion medium for 2–4 passages (P0–P4) before being cryopreserved.

NSCs were thawed in NMM supplemented with 10 ng/ml FGF2 on poly-ornithine and laminin-coated 6-well plates. The next day cultures were passaged once more with Accutase, replated at 50,000 cells/cm^2^ on poly-ornithine and laminin-coated surfaces (96-well plate for HTS (PerkinElmer, Belgium), 13 or 18 mm glass coverslips for immunofluorescence analysis (VWR, Belgium); 24 and 96 multiwell MEA plates (Multichannelsystems, Germany)). The next day FGF2 was withdrawn and cultures maintained for up to DIV90 (MEAs) with a medium change every 3–4 days.

To differentiate NSCs into astrocytes, we followed the protocol described by Shaltouki et al.^4^, with slight modifications. In brief, NSCs were re-plated at 50–60% confluence on poly-ornithine-laminin or Geltrex coated dishes in NMM supplemented with 20 ng/ml of FGF2. The next day, cells were switched to fresh NMM containing 10 ng/ml of Heregulin 1β, and 200 ng/ml of IGF-I analog (all from Peprotech). Medium was changed every other day and cells were passaged at least five to six times during the differentiation process, to DIV 90–100. From DIV100 onwards, astrocytes were cultured, expanded and cryopreserved in Astrocyte medium from ThermoFisher Scientific. The medium is composed of DMEM-high-Glucose w/ Sodium Pyruvate supplemented with 1X N2 and 10% FBS. To induce a resting-like quiescent phenotype, astrocytes were cultured from DIV100 onwards in serum-free medium NMM supplemented with 50 ng/ml FGF2. To use the astrocytes in specific biochemical assays, we switched culture conditions 24 h prior to use to Astrocyte medium devoid of FBS, but supplemented with 10 ng/ml HB-EGF (Peprotech).U+

### Astrocyte-conditioned media

Prior to preparing Astrocyte-conditioned media (ACM), astrocytes were cultured to 90% confluency on a 10-cm plate. Old FBS-containing medium was replaced with conditioning medium (50% neurobasal, 50% DMEM, glutamine, pyruvate, and penicillin–streptomycin) and astrocytes were cultured for an additional 24 h. Media were harvested, and dead cells and debris removed by centrifugation. The collected media was either used immediately or frozen at −80 (◦)C until needed.

### Western blot analysis

DIV100-DIV120 astrocytes were grown to 100% confluence and then were manually collected on ice and fresh-frozen cell samples were maintained at −80 °C until further processing. Samples were hydrolyzed in RIPA buffer (containing 50 mM Tris, 150 mM NaCl, 1% (vol/vol) NP40, 0.5% sodium deoxycholate (wt/vol), 0.1% SDS (wt/vol) complemented with protease inhibitors (Complete, Roche Diagnostics, pH 7.6). Protein concentrations were determined using the microBCA kit (Thermo Fisher Scientific) according to the manufacturer’s instructions. In brief, due to limited protein harvest, the entire protein sample was loaded/resolved for all lanes, on a Criterion XT 3–8% Tris-Acetate gel in XT Tricine Running buffer and run as previously described^13^. After running, samples were blotted to Nitrocellulose paper using the Trans-blot Turbo Transfer System (Biorad). Blocking was done for 1 h with 5% nonfat milk solution in PBST before incubation with primary antibodies; Dystrophin ab154168 (Abcam, rabbit) 1:2000 Gapdh ab128915 (Abcam, rabbit) 1:10,000. Protein bands were visualized using the Odyssey (Westburg, the Netherlands) after staining with IRDye 680TL (1:2000) and goat-anti-rabbit 926-68021 (Licor-Biosciences) (1:2000) labeled secondary antibodies.

### Quantitative real-time PCR

Total RNA was purified using the GenElute™ Mammalian Total RNA Miniprep Kit (Sigma-Aldrich) and ZR RNA MicroPrep (Zymo Research, USA). After concentration and integrity validation (NanoDrop 1000, Thermo Fisher Scientific, USA), cDNA was generated using 0.5–1 μg of RNA with SuperScript® III First-Strand Synthesis SuperMix for qRT-PCR kit (Invitrogen, USA). Real-Time qPCR was performed in technical triplicates on a ViiA™ 7 Real-Time PCR System with 384-well plate (Applied Biosystems, USA) with a Platinum® SYBR® Green qPCR SuperMix-UDG w/ROX (Invitrogen, CA, USA) and primers mix at final concentration of 250 nM. Gene expression (Cycle threshold) values were normalized based on the *GAPDH* (Glyceraldehyde 3-phosphate dehydrogenase) housekeeping gene and the Delta CT calculated.

### Immunofluorescence staining

Immunofluorescence staining experiments and image acquisition and analyses were performed essentially as described by Battacharyya et al.^5^ For non-high-throughput assays (such as neuron and astrocyte morphometry), cells grown on glass slides were fixed, permeabilized and dehydrated with 100% methanol for 5 min at −20 °C. Cells were rehydrated in BS3 cross-linker (Pierce, US) and incubated for 30 min at room temperature. Each well was then washed with 0.1 mM Glycine and then incubated overnight at 4 °C with appropriate primary antibody dilutions (along with the relative isotype controls, see Key resource table). The following day, cells were washed 3 times with PBS (+ 0.1% Triton-X-100 (Sigma Aldrich)) and incubated for 1 h at RT with fluorescently-labeled secondary antibodies diluted to 1:500 cells. The nuclei were counterstained with Hoechst dye (1:10,000 dilution) and mounted with ProLong® Gold Antifade Mountant (ThermoFisher Scientific).

### Microscopy, image acquisition, and high-throughput cell analysis

For wide-field imaging of immunofluorescent stainings, astrocytes were examined using an Axioimager.Z1 microscope (Carl Zeiss, Germany), equipped with an AxioCam MRc5 (bright field, Carl Zeiss) or a monochrome AxioCam Mrm camera (fluorescence, Carl Zeiss). Images for neuronal reconstruction were obtained using a Nikon C2 Eclise Ni-E Confocal confocal microscope (Nikon, Tokyo, Japan). To avoid non-specific bleed-through, each laser line was excited and detected independently. All images shown represent either a single confocal z-slice or z-stack. For quantification purposes, at least five independent fields per condition and per experiment were obtained.

For assessment of read through experiments on astrocytes, 10,000 DIV120 astrocytes were plated per well of a CellCarrier 96-well plate (PerkinElmer, UK) and allowed to attach for 24 h. Following treatment with Gentamicin (Sigma-Aldrich), G418 and PTC124 (Sanbio, Netherlands), they were fixed and stained with antibodies against dystrophin, α and β sarcoglycan (concentrations indicated in Key resource table) Cellmask (ThermoFisher Scientific) was used as a counterstain to facilitate segmentation and plasma membrane detection, allowing specific detection of surface localized staining of dystrophin. Images were acquired on an Operetta High Content Screening (HCS) System (Perkin Elmer, UK) equipped with climate control (37 °C, 5% CO2) using a 10X objective lens. Each experimental condition was assayed in at least triplicate wells and a minimum of 24 fields per well. Images were analyzed using Harmony software with PhenoLOGIC (PerkinElmer). Appropriate fluorescent channel filters used for image acquisition on the Operetta HCS imaging system. Using Cellmask to segment cells and outline plasma membrane, the algorithm can be specified to detect only surface localized staining of other markers such as dystrophin.

### Cell tracing and morphological analysis

2D neuronal tracings were generated in Neuronstudio (CNIC, Mount Sinai School of Medicine). Neuron tracings were exported in SWC file format, for compatibility with standard morphometric software tools. Multiple morphological parameters were extracted using the applications Neuronstudio, Simple Neurite Tracer, and L-Measure^6^. Quantification of the morphological properties of cells required two main steps: (i) extraction of each cell in the images from the respective background, a process called segmentation and (ii) calculation of several features describing complementary aspects of the geometrical properties of the cells.

Morphometric parameters of DIV120 astrocytes (grown to 60% confluency in order to trace individual cells) were analyzed using the image analysis software ImageJ. ImageJ was used to adjust the image brightness/contrast and images were saved as 8-bit tiff. Because of the varying contrast and superposition/juxtaposition of cells, and in order to ensure proper selection of valid cells against residual traces, the segmentation of the cells was performed. Each cell was subsequently processed in a fully-automated fashion in order to derive a broad set of morphological features including: perimeter (P) and area. Sholl analysis was performed with built-in plugin in ImageJ.

### Electrophysiological recordings: patch-clamp astrocytes and multielectrode arrays (MEA) neuronal network analysis

Electrodes for electrophysiological recording were pulled on a Flaming/Brown micropipette puller (Model P-87, Sutter Instrument, USA) from filamented borosilicate capillary glass (1.2 mm OD, 0.69 mm ID, World Precision Instruments, USA). The electrode resistances were 2–5 MΩ for whole-cell recording and 5–10 MΩ for cell-attached single channel recording. All recordings were performed at room temperature. Macroscopic currents are acquired in the voltage-clamp configuration using an Axon CV-4 headstage and Axopatch 200A amplifier (Molecular Devices, USA), low-pass filtered at 1 kHz, and digitized at 2 kHz using a DigiData 1322A (Molecular Devices, CA, USA). Single channel recordings were filtered at 1 kHz and digitized at 10 kHz. The series resistances were compensated at 90% level with the Axopatch 200A amplifier. Liquid junction potentials were nulled for each individual astrocyte. Membrane potential (Vm) was measured immediately after the establishment of whole-cell configuration. To examine the potassium uptake ability in astrocytes, the holding Vm was fixed at cell membrane potential under 3 mM K+ bath solution. By increasing the extracellular K+ from 3 mM to 12 mM and 60 mM, the induced inward K+ currents represent the ability of astrocytes to buffer potassium.

Neuronal activity was recorded using a multiwell multielectrode array (MEA) system (Multi Channel Systems, Germany). 10,000 NSCs from ND and DMD samples were plated in triplicates, on poly-L-ornithine and laminin and allowed to differentiate to neurons as described above. After DIV 60, NMM culture medium was transitioned to Brainphys^67^ (Stem Cell Technologies), supplemented with N2 and B27. Cells were fed once a week.

Spontaneous network activity from cortical cultures grown on MEA plates was recorded using software from multiwell multielectrode array (MEA) system. Recordings were performed using a band-pass filter with 10 Hz and 2.5 kHz cutoff frequencies. Spike detection was performed using an adaptive threshold set to 5.5 times the standard deviation of the estimated noise on each electrode^7^.

For assays wherein conditioned media, glutamate or specific inhibitors were tested, baseline recordings of 60 sec were made as above. Subsequently, stock concentrations of NMDA, Glutamate, CNQX, AP5, or PTC124 were diluted in 200 µL of media and added to the appropriate well such that the final concentration is reached, as indicated in the results (all from Sigma-Aldrich). DMSO was used as a vehicle control at 0.1% final concentration.

### Biochemistry and metabolism assays

#### Glutamate quantification

To evaluate the glutamate clearance capacity, DIV 120 astrocyte cultures grown in 24 well dishes to 90% confluency, followed by 500 μM Glutamate supplementation challenge. Media were collected from astrocytes (100 μL) at 0, 60, 120, and 180 min to evaluate the Glutamate remaining in the medium and stored at −20 °C until use. Glutamate concentration in the medium was determined according to kit instructions (Abcam, MA) and read in a 450-nm microplate reader. A standard curve was constructed in each assay using cell-free culture medium containing known concentrations of glutamate. The concentration of extracellular glutamate in the samples was estimated from the standard curve. Time point of 120 min was determined to be optimal and in linear range of detection and used for the rest of the measurements.

As a control for each experiment, serum-free medium was added to empty wells of a 24-well dish (no astrocytes) and processed together with the astrocytes, i.e. 120 min in the incubator until sample collection. The assay normalization was based on nuclear staining quantification of DAPI, performed after the last collection of media. DAPI analysis was performed using ImageJ.

DIV120 Astrocytes grown to 90% confluence were washed once with PBS and the replenished with serum-free astrocytes, growth factor-free astrocyte medium. After 12 h, the medium was collected and glutamate concentration measured, as described for glutamate clearance assay.

#### Nitric oxide measurement

We assessed intracellular NO with DAF-2DA, a diaminofluorescein-2 diacetate fluorescence probe (10 μM). DIV120 astrocytes grown to 90% confluence were incubated with NMM containing DAF-2AM (ThermoFisher Scientific) for 60 min at 37 °C followed by a 30-min post-incubation period in NMM without the fluorescent indicator. NO production analysis was carried out using an Olympus IX71 widefield microscope system (Olympus) with excitation and emission wavelengths of 460–495 nm and 510–550 nm, respectively^10^. We recorded images using imageJ’s micromanager- Hamamatsu ORCA-Flash high speed camera (Hamamatsu Photonics) and measured the increases in the green fluorescence intensity.

#### Mitochondrial ROS stress test

Similar to NO measurements, we examined general ROS levels and mitochondria-specific-superoxide generation by fluorescence microscopy (Olympus IX71 widefield) using fluorescent probe CM-H2DCFDA (5 μM) and MitoSOX (5 μM), respectively (both from ThermoFisher Scientific) in DIV120 astrocytes grown to 90% confluency. We incubated cells with respective probe at 37 °C for 30 min and then recorded change in fluorescence intensity from vehicle treated baseline.

#### Calcium imaging

Calcium flux studies were carried out by plating DIV120 astrocytes on glass bottom microwell dishes (MatTek, Germany) and allowed to reach 90% confluence. Next, cells were incubated in Tyrode’s solution (in mM: 129 NaCl, 5 KCl, 2 CaCl2, 1 MgCl2, 25 HEPES, 30 Glucose, pH 7.4) supplemented with 5 μM Fluo-4 (ThermoFisher Scientific) and 0.02% Pluronic F-127 (Sigma) for 45 min at RT and 5% CO2 in the dark. Fluorescence microscopy was performed using an Olympus IX71 widefield microscope system (Olympus, Japan) with a ×20 objective, using time-series frames with an interval of 2 s, using Hamamatsu ORCA-Flash high speed camera (Hamamatsu Photonics, Belgium). For each biological replicate, 10–20 cells were measured. Traces in the graphs represent the normalized average fluorescence intensity change over time.

### Statistics

All data represent a minimum three or more independent biological replicates (i.e., cultures at different passages) (unless otherwise specified in the figure legends) ± standard error of the mean (SEM). Comparisons between two groups were analyzed using unpaired or paired two-tailed Student’s *t*-test. *P*-values < 0.05 were considered significant (*). Data are shown as mean, and error bars represent standard error of mean of a minimum three independent experiments. For patch-clamp data, a Dunnett’s multiple comparison test and Sidak’s multiple comparisons test were used to compare respectively two or three groups as patch clamp data were not normally distributed. Results were plotted and analyzed using GraphPad Prism 6 software.

### Study approval

The protocol to obtain blood or fibroblasts DMD subjects and normal donors was approved by the Ethics Committee of the University Hospitals Leuven (Clinical Trial Center (CTC), UZ Leuven, Campus Gasthuisberg #S55438, Jul 31, 2013). Consent was obtained from the DMD subjects’ guardian and an assent from the DMD subject himself to donate 5–10 cc of blood; while for D2, a skin biopsy was obtained.

## Results

### Generation of DMD subject-specific iPSCs, neuronal progeny and astrocytes derivatives

We generated human iPSC lines from peripheral blood (*n* = 5) and fibroblasts (*n* = 1) of DMD subjects with mutations located at different loci along the length of the *DMD* gene^45,46^using Sendai viral vectors, encoding the canonical ‘Yamanaka factors’. The characteristics of the six DMD subjects are summarized in Supplementary Table 1. For each subject, we generated at least two different clones. These clones were expanded, characterized and banked for future utilization (Supplementary Table 2). All clones were demonstrated to be pluripotent based on flow cytometric analysis (Fig. S1A), immunostaining for pluripotency markers (Fig. S1B), evaluation of teratoma (Fig. S1C), hPSC scorecard/embryoid body (EB) formation (Fig. S1D i–iv), pluripotency gene expression (Fig. S1D v–vii). Elimination of Sendai viral-vector was demonstrated by PCR (Fig. S1D (viii), and genome integrity by array comparative genomic hybridization (data not shown). In some, but not all, experiments of both clones from a single subject were tested (for all D1 = D1.01, D1.02).

DMD iPSCs and Normal Donor (ND) PSCs (the human embryonic stem cell (ESC) line H9, and three different iPSCs) (see Supplementary Table 2) were differentiated towards cortical neuronal progeny using methods adapted from Shi et al.^47^, as depicted in Fig S2A. Neural stem cell (NSC) rosettes, differentiated from ND and DMD iPSCs on day in vitro 14 (DIV14) were morphologically indistinguishable (*n* > 4 per cell line). NSC rosettes stained uniformly positive for NESTIN and paired-box 6 (PAX6) (Fig S2B), and contained a similar fraction of BLBP (iii) and FOXG1 (iv) positive cells (Fig S2B). They also expressed similar mRNA levels for forebrain genes, such as Orthodenticle Homeobox 2 (*OTX2*), and Forkhead box protein G1 (*FOXG1*) (Fig S2B v and vi). When differentiated to DIV30 neuronal progeny, cells stained uniformly positive for the neural marker beta tubulin-3 (B3T, TUJ1), and >60% of the cells stained positive for the deep-layer cortical neuronal marker, T-box brain 1 (TBR1) (Fig S2C). Both ND (i) and DMD (ii) DIV30 neurons contained a similar fraction of TBR1 positive cells (Fig S2C iii). ND and DMD NSC progeny expressed similar levels of transcripts for lower layer cortical neuronal genes, including TBR1 (iv), B-Cell CLL/Lymphoma 11B (*BCLL11B* also named *CTIP2*) (v) and *vGLUT1* (vi). Further functional characterization of DIV90 neurons (Fig S2D) by multielectrode array (MEA) measurements did not show any consistent changes between ND and DMD neuronal progeny. Absence of obvious defects in DMD cortical neuronal progeny is in line with the fact that DMD neuronal progeny did not express detectable levels of major dystrophin isoform transcripts, except very low levels of the known, ubiquitous *Dp71* isoform (Fig S2E).

**Fig. 1:**
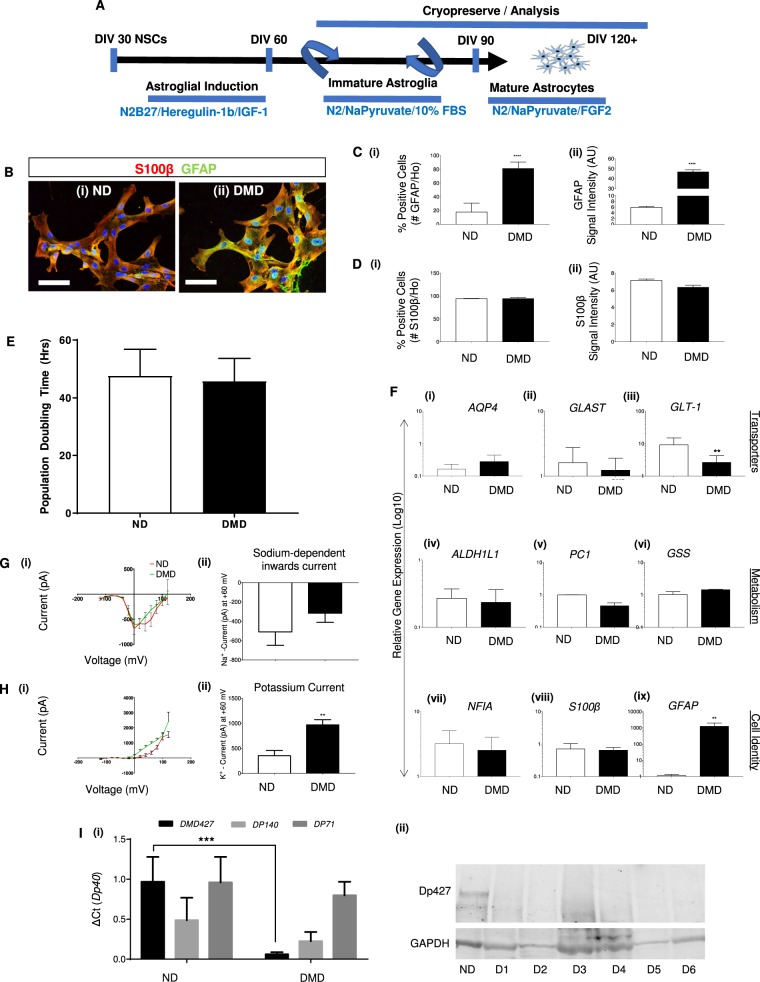
DMD iPSCs successfully differentiate into neurons and astrocytes. **a** Schematic of generation and long-term culture of human astrocytes with immature, and mature (quiescent) phenotypes from hPSCs. **b** Representative Immunostaining of ND (ChiPSC6B and Bj1-iPSCs) and five DMD lines (D1, D2, D3, D5, and D6) at DIV120 astrocytes stained with antibodies against S100β (Red), GFAP (Green) and Hoechst (blue). Scale bar represents 100 μm. Staining was done for all lines, *n* = 4 biological independent astrocyte derivation experiments, starting from iPSCs cultures. **c** Quantification of GFAP expression (i) in ND (ChiPSC6B and Bj1-iPSCs) and DMD (D1, D2, D3, D5, D6) DIV120 PSC astrocyte progeny. Relative Intensity of fluorescent signal, normalized to Hoechst (Ho) positive nuclei, for GFAP (ii). *n* = 4 biological replicates, for each independent experiment; number of positive cells and intensity determined in technical replicates of 24 wells of a 96-well plate on the Operetta system. **d** Quantification of S100β expression (i) in ND and DMD DIV120 PSC astrocyte progeny. Relative Intensity of fluorescent signal, normalized to Hoechst (Ho) positive nuclei, for S100β (ii). *n* = 4 biological replicates, for each independent experiment; number of positive cells and intensity determined in technical replicates of 24 wells of a 96-well plate on the Operetta system. **e** Cell doubling period. Y-axis represents cell doubling time in hours, starting from initial plated density of 100,000 cells per well of a standard 12-well tissue culture plate. *n* = 4 biological replicates, for each independent experiment; number of cells were determined in technical replicates of three wells of a 12-well plate, at each time point. **f** Transcript levels of a selected number of transporters (i–iii), metabolism genes (iv–vi), and cell identity genes in ND and DMD DIV120 astrocytes. Levels shown as relative gene expression, CT value of gene of interest divided by CT value for housekeeping gene (GAPDH). *n* = 4 biological replicates, one column of 384-well qPCR plate (i.e. 16 wells) was used as technical replicates for each of *n* = 4 runs. **g** Representative traces of Sodium current-voltage relationships (IV curves) for ND and DMD DIV120 astrocytes (i). Traces representative of ND (ChiPSC6B, 20 cells) and DMD lines (23 cells) (ii). Mean ± SEM of current-Voltage ratio at +60 mV, for ND and DMD Astrocytes. **h** Representative traces of Potassium current-voltage relationships (IV curves) for ND and DMD DIV120 astrocytes (i). Traces representative for the ND (ChiPSC6B, 20 cells) and the DMD lines (23 cells) (ii). Mean ± SEM of current-Voltage ratio at +60 mV, for ND and DMD Astrocytes. **i** qRT-PCR analysis for dystrophin isoforms expression in ND and DMD DIV120 astrocytes (i). Western blot of dystrophin isoform Dp427 in DIV120 ND (ChiPSC6B) astrocytes and all DIV120 DMD astrocyte lines. GAPDH was included as the loading control (ii). All quantitative data (except the Western blot in I, where only one blot was performed) is shown as Mean ± SEM of *n* = 4 biological replicates, ND (ChiPSC6B and Bj1-iPSCs) and 5 DMD lines (D1, D2, D3, D5, D6). All analyses based on Student’s *t*-test, *P < 0.01, **P < 0.001; ***P < 0.0001, ****P < 0.00001

We next differentiated DMD and ND PSCs into astroglial progenitors and subsequently into mature astrocytes using a protocol modified from Shaltouki et al.^48^, as depicted in Fig. 1a. Astroglial differentiation was initiated by exposing NSCs to heregulin-1b and insulin-like growth factor 1 (IGF1) (DIV30-60), followed by culture with 10% fetal bovine serum, N2 supplement and sodium pyruvate (DIV60-90), and FGF2 with N2 and sodium pyruvate (DIV90-120). Initial immunostaining for the classical astroglial identity markers S100 calcium-binding protein B (S100β) and glial fibrillary acidic protein (GFAP), of ND (i) and DMD (ii) PSC astrocyte progeny on DIV120 demonstrated that both cell types generated nearly 100% astroglial cells (Fig. 1b). When re-examined on DIV120, significantly more DMD progeny stained positive for GFAP (Fig. 1c i), and the GFAP signal intensity (ii) was significantly higher in DMD compared with ND astrocytes (Fig. 1c). However, the frequency (Fig. 1d (i)) and intensity (Fig. 1d ii) of S100β positive cells was similar in DMD and ND astrocyte progeny. In addition, the proliferation rate of both ND and DMD astrocyte progeny was similar (Fig. 1e). Astrocyte differentiation was further demonstrated by qRT-PCR for transcripts of a select number of astroglial specific genes on DIV120 (Fig. 1f i–ix).

Initial basal electrophysiological examination of both DIV120 ND and DMD astrocytes demonstrated unremarkable difference in cell capacitance (Fig. 3SA (i)), comparable resting membrane potential (Fig. 3SA (ii)) and a typical sodium dependent, inwards current-voltage relationships curves (Fig. 1g). However, the ion-gated potassium channel functionality was disturbed in DMD astrocytes, in line with previous reports that found an association of inwardly rectifying potassium channel (Kir4.1) with dystrophin, via the α-syntrophin linker protein^49,50^(Fig. 1h); representative current trace for ND and DMD/D2 is shown in Fig. S3B (i)-(ii). Further validation of reduced Kir4.1 expression followed from immunostaining and quantification analysis (Fig. S3C (ii)).

Lastly, DIV120 DMD astrocytes, unlike ND cells, did not express the full-length isoforms of dystrophin depending on the exact location of the mutation: for instance, astrocytes from DMD-D1, comprising of a large deletion, did not express any appreciable levels of either *Dp427* or *Dp140* transcripts; DMD-D2 astrocytes harboring a point mutation in exon 36, did not express *Dp427* transcripts while *Dp140* transcripts levels were decreased. (Fig. 1i (i)). Only *Dp71* expression seemed universally preserved in all astrocytes. This trend was confirmed by western blotting (Fig. 1i (ii)) where the full-length isoform Dp427 protein band was visible in ND astrocytes, but absent in all DMD mutant astrocytes.

These studies demonstrated that, although we could not detect obvious defects in cortical neuronal and astrocytic progeny from DMD iPSCs, DMD astrocytes appeared to display a more activated state compared to ND astrocytes^51^.

### Dystrophic astrocytes manifest features typically seen in DMD myocytes

**Fig. 2:**
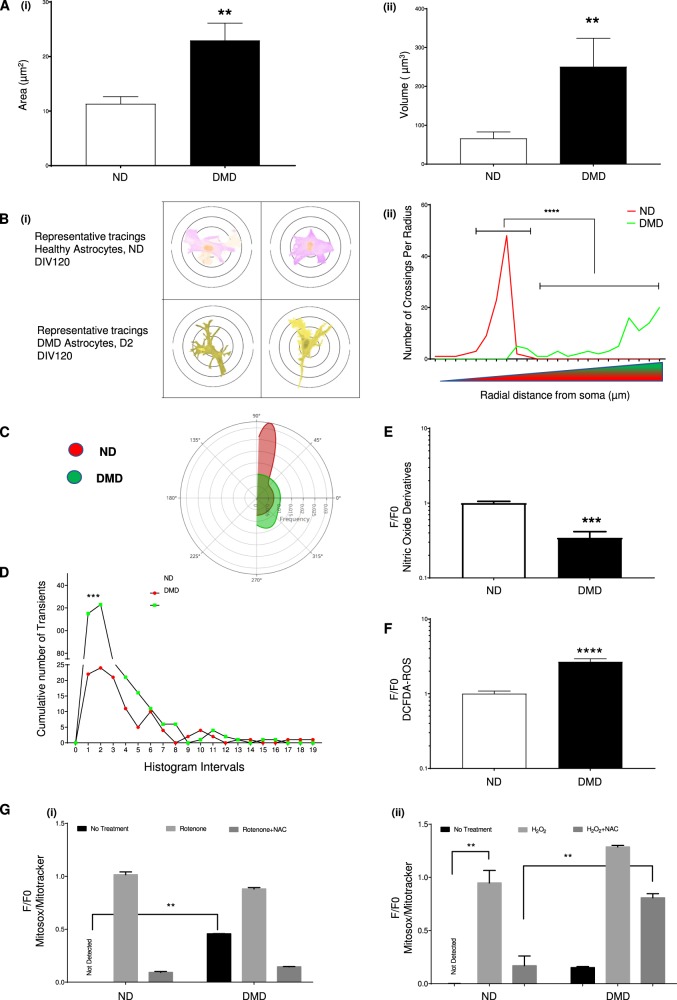
Loss of dystrophin from astrocytes presents defects typically also seen in DMD myocytes. **a** Morphometric parameters to describe the cytoskeletal organization in ND (ChiPSC6B, Bj1-iPSCs, H9-ESCs) and DMD (D1, D2, D3, and D5) astrocyte progeny. Area (i), volume (ii). *n* = 4 biological replicates, for each independent experiment; morphometric parameters were quantified in technical replicates of 24 wells of a 96-well plate on the Operetta system. **b** Schematic illustration of classical Sholl analysis with radii increasing in 10 μm increments to quantify process lengths. Representative traces of ND (ChiPSC6B) and DMD (D2) DIV120 astrocytes (i). Mean number of intersections ND (ChiPSC6B, Bj1-iPSCs, H9-ESCs) and DMD (D1, D2, D3, and D5) astrocyte makes with concentric circles of increasing radii (ii). Data shown as mean ± SEM of *n* = 4 biological independent experiments, technical replicates of *n* > 100 astrocytes for each genotype per experiment. **c** Radial histogram of the directionality of ND and DMD DIV120 astrocyte progeny (the local gradient orientation of directionality). Shown is BJ1 for ND Astrocytes and D2 for DMD Astrocytes. *n* = 4 biological replicates, for each independent orientation quantification, five random images per well (24 wells per cell line) were obtained using an Operetta system in 96-well plates. **d** Quantification of calcium flux in ND and DMD DIV120 astrocytes, measured as spontaneous and transient sparks of increased fluo-4 intensity at a given time (mean), normalized to those at baseline (time 0). Red: ND (ChiPSC6B, Bj1-iPSCs, H9-ESCs); Green: DMD (D1, D2, D3, and D5). Data shown as mean ± SEM of *n* = 4 biological independent experiments, technical replicates of *n* > 100 active astrocytes for each genotype per experiment. **e** Quantification of Nitric Oxide (NO) production in ND (ChiPSC6B, Bj1-iPSCs, and H9-ESCs) and DMD (D1, D2, D3, and D5) DIV120 astrocytes by DAF-2AM staining. Green fluorescence (F) marked cells that produce NO, as such change of fluorescence intensity from baseline (F0), over time (F/F0) reflected the levels of NO in the medium. *n* = 4 biological replicates, for each independent measurement, five fixed regions per image per well (24 wells per cell line) were obtained on Operetta system in 96-well plates at multiple time points. **f** Relative quantification of ROS production in ND (ChiPSC6B, Bj1-iPSCs, and H9-ESCs) and DMD astroglia (D1, D2, D3, and D5) DIV120 astrocytes using DCFDA-CM derivatives. Shown is the quantified change in green fluorescence intensity from baseline (F0), over time (F/F0) marking cells that undergo oxidation. *n* = 4 biological replicates, for each independent measurement, five fixed regions per image per well (24 wells per cell line) were obtained on Operetta system in 96-well plates at multiple time points. **g** Confluent ND (ChiPSC6B, Bj1-iPSCs, and H9-ESCs) and DMD (D1, D2, D3, and D5) DIV120 astrocyte monolayers were treated with DMSO, Rotenone (i) and 10 μM H2O2 (ii) or ROS inducer plus *N*-Acetyl-Cysteine (NAC); Average ratio of Mitosox Red intensity to Mitotracker Green intensity, used to normalize for total cellular mitochondria. *n* = 4 biological replicates, for each independent measurement, five fixed regions per image per well (24 wells per cell line) were obtained on Operetta system in 96-well plates at multiple time points. All quantitative data is shown as Mean ± SEM of *n* ≥ 4 biological replicates. All analyses based on Student’s *t*-test, *P < 0.01, **P < 0.001; ***P < 0.0001, ****P < 0.00001. Except for F, Dunnett’s multiple comparison test and Sidak’s multiple comparisons test were used

Dystrophin, as part of a scaffolding complex, is well known to affect morphological and cytoskeletal features of muscle cells. Therefore, we examined if loss of dystrophin from astrocytes might affect their morphological characteristics. On DIV120, morphometric analysis of phalloidin stained F-actin cytoskeleton in astrocyte progeny demonstrated an increased cell area (Fig. 2a i) and a significantly larger cell volume for DIV120 DMD astrocyte progeny (Fig. 2a ii). This was further confirmed by a Sholl analysis, revealing a greater degree of branching of DMD compared to ND astrocyte progeny (Fig. 2b i and 2B ii), which might be due to the lack of dystrophin protein. On DIV120, we also noted that DMD astrocyte progeny displayed a more unorganized cell monolayer with no preferred polarity/cell orientation, whereas ND astrocytes clearly aligned in a preferential manner (Fig. 2c).

Next, we determined if, aside from the differences in DMD astrocyte morphology, other defects described in DMD skeletal myofibers, such as aberrant Ca^+2^ handling^52^, decreased NO production, increased ROS accumulation^53,54^, and increased mitochondria-specific ROS production^55,56^, were also found in DMD astrocyte progeny.

Astrocytes are known to respond to synaptic release of glutamate and ATP through increased intracellular Ca^+2^ levels. Using the Fluo-4 dye, we assessed spontaneous astrocyte Ca^+2^ cycling in DIV120 DMD and ND PSC-derived astrocytes. While spontaneous Ca^+2^ transients, identified by change in Fluo-4 fluorescence intensity, were observed in DMD and ND astrocytes, the transient frequency was significantly elevated. That is, in DMD compared to ND astrocyte progeny (Fig. 2d) the frequency and amplitude of transient rise and oscillations in cytoplasmic calcium concentration over a given period of time. Moreover, DMD astrocytes were observed to be more robustly able to respond to a 1-mM ATP biochemical stimulus with a larger amplitude than ND astrocytes (Fig S3D).

Further metabolic studies involving calcium, ROS and glutamate measurements, indicated that DIV120 DMD astrocytes contained significantly less NO, as assessed by the formation of the highly fluorescent triazolofluorescein (DAF2AM) derivative from its non-fluorescent original diaminofluorescein (DAF) state upon NO binding (Fig. 2e). Moreover, significantly higher levels of cellular ROS were detected in DMD than in ND astrocytes, as shown by DCFDA staining (Fig. 2f). Finally, we observed reduced ROS buffering capacity in DMD astrocytes. A significantly greater resting mitochondrial ROS level was observed, as well as an increased susceptibility of DIV120 DMD astrocytes to mitochondrial stress, demonstrated by the ratio between mitochondrial ROS levels (MitoSox) and ROS-independent, generic mitochondrial dye (Mitotracker) following addition of either Rotenone (Fig. 2g (i)) or direct oxidative stress by H_2_O_2_ application (Fig. 2g (ii)). Although Rotenone toxicity, via inhibition of mitochondrial respiratory chain complex I, could be blocked by the anti-oxidant *N*-acetyl-L-cysteine (NAC), a putative free-radical scavenger, in both ND and DMD astrocytes (Fig. 2g (i)), NAC significantly decreased mitochondrial ROS only in H_2_O_2_ treated ND astrocytes, while DMD astrocytes remained highly ROS positive (Fig. 2g (ii)).

Thus, DMD astrocytes appeared to have a defective cell cytoskeleton, and displayed aberrant Ca^+2^ handling, decreased NO, elevated ROS levels and increased mitochondrial susceptibility to ROS- inducing agents, such as Rotenone and H_2_O_2_, all hallmarks of dystrophin-deficient DMD myofibers.

### RNA sequencing identified dysregulated pathways in DMD astrocytes that otherwise maintain astroglial homeostasis

**Fig. 3:**
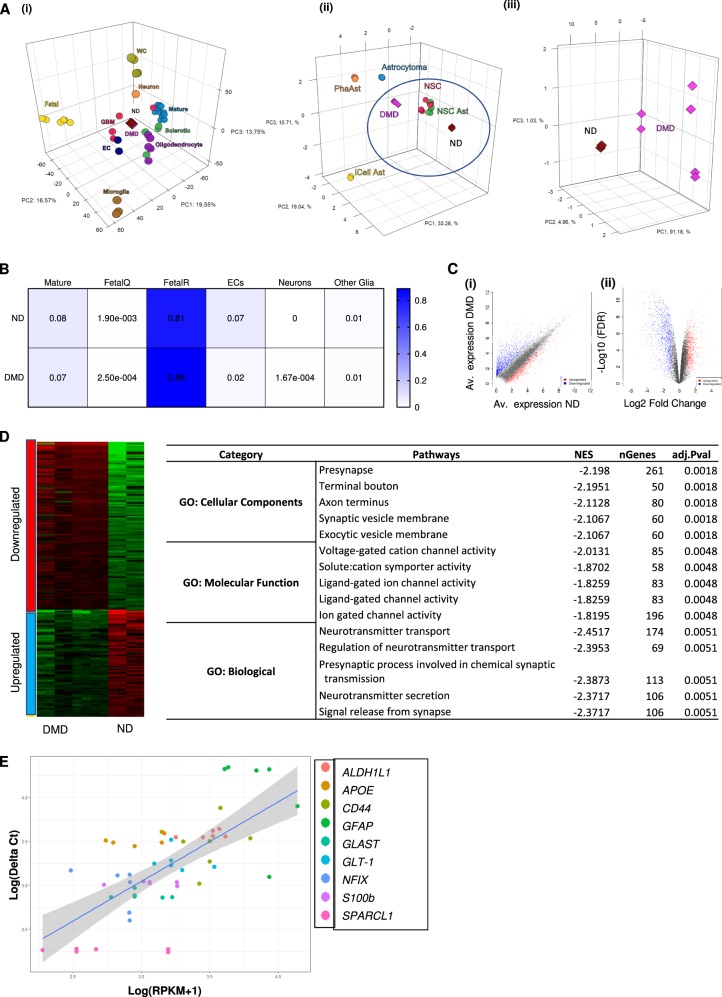
Global gene expression profiling demonstrates significant dysregulation among solute carrier family of genes and processes involving cell membrane and mitochondrial membrane in DMD astrocytes. **a** 3D PCA plots of ND/DMD astrocyte profiles as compared to published transcriptomes of CNS cell types. unbiased clustering of ND (Bj1-iPSC) and DMD (D1, D2, D3, and D6) DIV120 astrocytes using t-SNE correctly shows their distinct clustering in an in vivo context of multiple CNS cell types (i), in an in vitro setting of other published PSC-derived astrocyte and glioma cell transcriptomes (ii), and between ND and DMD astrocytes from the current study (iii). **b** CIBERSORT RNA deconvolution of all transcripts detected at logFC > 1. Purified astrocytes are not contaminated by other lineage cells as indicated by mRNA fractional composition analyses, confirming the astrocytic identity of profiled samples. Also confirmed is the fetal nature and an actively proliferating phenotype. **c** Scatterplots of expression levels of all DEGs in ND versus DMD astrocytes. Each point represents an individual gene and all genes differentially expressed in DMD samples with an FDR of 0.05 or less (i); which are depicted in red = Upregulated and Blue = Downregulated. Volcano plot of significant differential gene expression patterns in ND versus DMD astrocytes, providing *P* values and fold change to illustrate correlation of gene expression (ii). **d** Heatmap overview of all altered pathways. Gene Ontology (GO) Pathways analysis using differentially expressed genes in DMD astrocytes show the pathways most strongly altered when dystrophin is absent. Adjusted *p*-values, number of relevant genes and normalized enrichment scores (NES) are indicated in the table. **e** Quantitative RT-qPCR validation of RNAseq differential expression analysis. Technical validation of RNAseq differential expression analysis using RT-qPCR showing high degree of correlation between log2-fold change differences from RNAseq and RT-qPCR data for nine different genes. Abbreviations: WC = Whole Cortex, Fetal = Fetal astrocytes, Mature = Mature Astrocytes, GBM = Glioblastoma, Sclerotic = Sclerotic astrocytes, EC = Endothelial Cells. PhaAstrocytes = Cultured primary human astrocytes, NSC = PSC- derived Neural Stem cells (NSCs), NSC Ast = PSC-derived astrocytes via NSCs intermediate, iCell Ast = commercially available iPSC-derived astrocytes. Healthy = ND DIV120 astrocytes, DMD = DIV120 DMD astrocytes

To gain further insights in differences between DMD and ND astrocyte-progeny we performed RNA sequencing (RNAseq) of 2 biological replicates of DIV120 astrocyte progeny from ND ChiPSC6B-iPSC and of 4 DMD iPSC harboring a spectrum of known mutations i.e. a large exon deletion (D1-Δ49-52, D3-Δ16-17, D4-Δ51-55 and D5-Δ46-51) and a point mutation in exon 36 (D2-c.4996C>T). We first compared the transcriptome of ND and DMD PSC-astrocytes with the published transcriptional signature of primary brain cells (Fig. 3a (i)). The iPSC-derived astrocytes clearly clustered far from other in vivo neuronal cell transcriptomes, except astrocytes. As expected, they were instead positioned between fetal and mature in vivo astrocytes. Moreover, when compared to transcriptomes obtained from cultured glial (and glioma cells), as expected, PSC-astrocytes derived in this paper, regardless of genotypic category, associated closer to NSC derived in vitro progeny than cultured primary isolated glial cells (ii). Lastly, compared between themselves, the ND and DMD astrocytes segregated away from each other (iii). CIBERSORT based deconvolution demonstrated that the transcript profiles of all PSC-derived astroglial progeny most closely resembled those found in cultured fetal astrocytes, with a less significant contribution of mature-astrocyte-like transcripts, and negligible transcripts associated with other neuronal cell types (Fig. 3b).

Subsequently, differential gene expression analysis was performed using a web-based R-dependent application, iDEP. A total of 13,755 genes were mapped to the reference genome. We found that 1055 genes were significantly upregulated and 1480 genes significantly downregulated in DMD compared to ND astrocyte progeny (Fig. 3c). To explore dysregulated pathways and gene set enrichment, we used built-in scripts from iDEP and Ingenuity pathway analysis (IPA), respectively. When comparing ND and DMD astrocyte progeny, key pathways that were altered included: presynapse cellular components, molecular activity of ligand-gated ion channel and most importantly, neurotransmitter regulation around the synaptic space (Fig. 3d). Moreover, at a systems level, the differentially expressed genes underline coherent alterations in groups of co-expressed genes, as detected via the weighted gene co-expression network analysis (WGCNA) algorithm (Fig. S4A), which revealed that the differentially expressed gene set is composed of four major modules. qRT-PCR confirmed the differential expression of genes identified by RNAseq between DMD and ND astrocyte progeny (Fig. 3e). Thus, RNAseq indicated that glutamatergic synapse processes, including neuro/glio transmission, amino acid transport across membrane in DMD astrocytes and synapse regulation were disturbed transcriptionally. The Turquoise and Blue modules identified by WGCNA also highlighted pathways involved in synaptic transmission and synaptic extracellular matrix, respectively (Fig. S4B). We also investigated if the deranged gene expression in DMD astroglial progeny converged on common biological processes associated with risk for ASD, using a set of 155 ASD genetic risk candidates from the Simons Foundation Autism Research Initiative (SFARI) AutDB database^57^. Among the dysregulated genes in the dystrophic transcriptome we observed a strong cross-disorder transcriptomic correlation with ASD-associated genes (*P* value < 0.01) (Fig. S4C (i)), which includes genes identified in previous genetic studies as well as genes investigated based on prior neurobiological hypotheses for ASD. More importantly, gene expression of the key regulator of glutamate and glutamine homeostasis, astrocytic glutamate uptake/release and the glutamate–glutamine shuttle is glutamine synthetase (*GLUL*)^58^, was decreased in DMD group, as shown in (Fig. S4C(ii)). In the context of shared gene families, the two transcriptomes also showed convergent pathways associated with elements of axonal guidance signaling and GABA/Glutamate receptor signaling, among other neuro-metabolic clusters (Fig. S4D).

### Dysfunctional glutamate handling in DMD astrocytes causes structural and physiological impairment of neuronal cells

Glutamate affects neurite outgrowth^59–61^ aside from exerting an excitatory role in the mammalian CNS^62^. As astrocytes are responsible for glutamate homeostasis in the CNS, we next assessed glutamate production and uptake by DMD vs. ND astrocytes, and the effect thereof on neurite outgrowth and electrophysiological characteristics of ND cortical neurons.

**Fig. 4:**
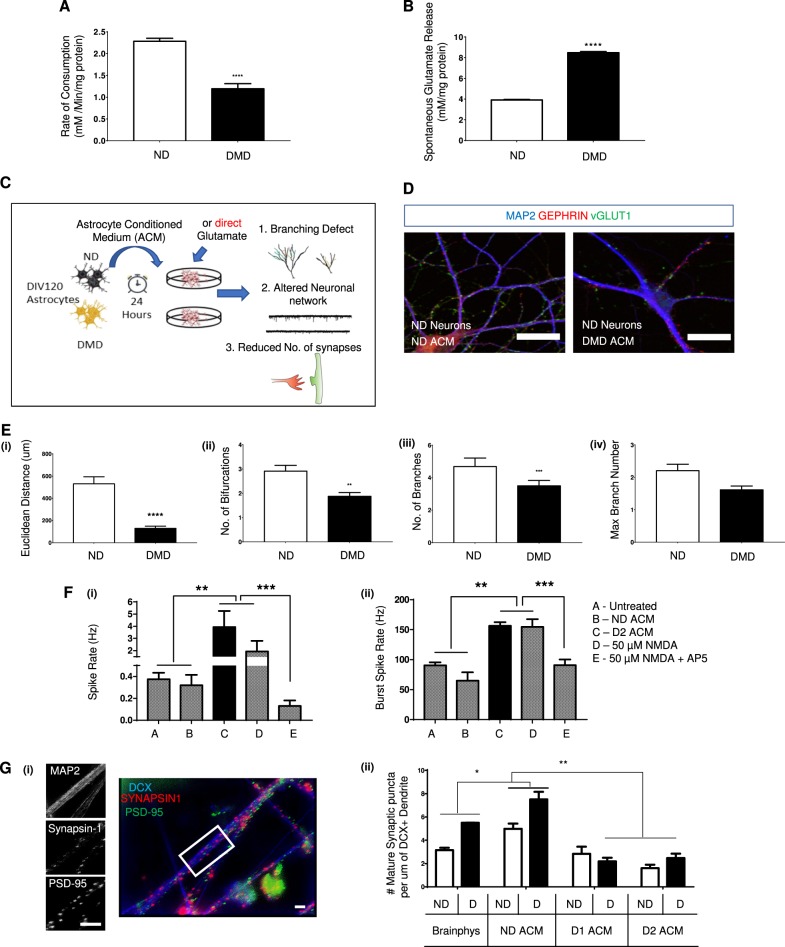
Excessive glutamate in DMD ACM is the primary mediator of abnormal neuronal development. **a** Change in the level of free glutamate in medium 180 min after incubating ND (ChiPSC6B, Bj1-iPSCs) and DMD lines (D1, D2, D3, and D5) DIV120 astrocytes with known concentrations of glutamate at time zero. **b** Net accumulation of glutamate in supernatants of ND (ChiPSC6B, Bj1-iPSCs) and DMD (D1, D2, D3, and D5) DIV120 astrocytes over 24 h period. **c** Experimental setup to study effect of glutamate-rich DMD astrocyte-conditioned medium (ACM) on the growth and functionality of ND neurons. **d** Representative images of dendrite branching complexity of DIV30 ND neurons cultured with either ND (ChiPSC6B) or DMD-D2-ACM for 12 h. (Scale bar, 100 μm). Immunostaining for MAP2 in Blue, GEPHRIN in Red and vGLUT in Green. **e** Quantification of different neuroanatomical parameters acquired from 2D traced digital reconstruction of neurons using the ImageJ software following incubation of DIV30 ND cortical neurons (ChiPSC6B) 12 h with ND or DMD ACM. Shown is Euclidean distance (ED) (i), number of bifurcation junctions (ii) number of branches (iii), and branch number (iv) (Mean ± SEM of ≥4 biological replicates, ACM from 2 ND (ChiPSC6B, Bj1-iPSCs) and 4 DMD lines (D1, D2, D3, and D5), *n* > 50 neuron tracings for each). **f** Multielectrode array (MEA) recordings of ND (KSF-16-025) DIV 90 cortical neurons were made to determine spike rate (i) and the burst spike rate (ii) upon various treatment conditions. Neurons with DMSO (Basal), neurons following a 60-min incubation with ND (ChiPSC6B) or DMD (D2) ACM. **g** Evaluation of synaptic density of ND [60] and DMD (D1, D2) DIV60 neuronal network with and without ACM from DIV120 ND (ChiPSC6B) and DMD (D1, D2) DIV120 astrocytes. The number of mature (colocalized) synaptic sites was quantified by colocalization of Synapsin-1 and PSD-95 immunopositive puncta per µm dendritic length (identified by DCX staining). (i) Representative image. (ii) Quantification of synapses in ND and DMD DIV60 neurons following addition of optimal Brainphys medium^67^, or ND (ChiPSC6B) or DMD (D1 and D2) ACM. ND and D here refer to the genotype of DIV60 cortical neurons. All quantitative data is shown as mean ± SEM of *n* ≥ 4 biological replicates. All analyses based on Student’s *t*-test, *P < 0.01, **P < 0.001; ***P < 0.0001, ****P < 0.00001. Except for G (ii), Dunnett’s multiple comparison test and Sidak’s multiple comparisons test were used

We assessed glutamate concentrations in medium conditioned by ND and DMD astrocytes (ACM) for 24 and 48 h using a colorimetric assay. Following a challenge of the cultures with a fixed amount of exogenous glutamate, DIV120 DMD astrocytes removed significantly less glutamate from the ACM compared to ND astrocytes, (Fig. 4a). In addition, we measured glutamate accumulation in DIV120 ACM in the absence of exogenous glutamate addition. This demonstrated that unlike ND astrocytes, DMD astrocytes spontaneously released glutamate in the ACM (Fig. 4b). Thus, glutamate production and glutamate uptake appeared defective in DMD astrocytes.

We performed a set of experiments to address the repercussion of the excess glutamate in DMD ACM on neurons (summarized in Fig. 4c). We assessed neurite outgrowth of DIV30 ND iPSC (ChiPSC6 line) derived cortical neuronal progeny when cultured with 50% ACM from DIV120 DMD or from ND astrocytes. We prepared low-density DIV30 cortical neuron progeny cultures to visualize individual neurons, followed by staining with antibodies against *microtubule associated protein 2* (MAP2), presynaptic protein inhibitory synapse (GEPHRIN), and the excitatory presynaptic protein, vGLUT1, 12–14 h following incubation with ND and DMD ACM (Fig. 4d). Neurite outgrowth and dendritic complexity was quantified using 2D traced digital reconstruction of neurons in ImageJ. DMD ACM caused a significant decrease in neurite complexity, leading to significantly decreased Euclidean distance (Fig. 4e i), reduced number of bifurcation junctions (Fig. 4e ii) and less branches compared to neurons treated with ND ACM (Fig. 4e iii). However, the maximum branch number (Fig. 4e iv) was not significantly affected by DMD ACM.

To prove that excess glutamate in DMD ACM was responsible for the aberrant dendritic phenotype, we first added 0–1000 μM glutamate to ND ACM and re-examined the dendrite formation of ND iPSC neural progeny (Fig. S5A). The dendritic morphology of neurons treated with ND ACM containing increasing concentration of glutamate, phenocopied, at a concentration of 1 µM glutamate, what we observed following addition of DMD ACM. As expected, addition of NMDA agonists to ND ACM triggered dendritic deformities (Fig. S5B (i)). Tetrodotoxin (TTX) was used as positive control for neuronal toxicity (Fig. 4f i). When the specific NMDA receptor antagonist D-(−)-2-amino-5-phosphonopentanoic acid (AP-5) was added to DMD ACM, we could completely block the effects of DMD ACM on neurite outgrowth (Fig. S5B (ii)). This was not the case when we used the α-amino-3-hydroxy-5-methyl-4-isoxazolepropionic acid receptor AMPA/kainate receptor (CNQX) inhibitor alone (Fig. S5B(ii)). Moreover, in combination with AP-5, CNQX prevented the neuroprotective effect of AP-5 alone. Thus, defective function of the NMDA receptor in DMD astrocytes is responsible for the excess glutamate in DMD ACM, causing neurite outgrowth defects.

To investigate the effect of DMD ACM on the electrophysiological response of ND neural networks, we plated ND iPSC-derived cortical neurons in 96-well MEA plates and recorded spontaneous extracellular field activity at DIV90 with or without ACM and other pharmacological treatments, added for 30 min. In addition, agonist and antagonist pairs, also described above, were applied acutely to neurons once baseline activity was recorded for 60 sec. NMDA application increased both the spike and burst frequency, as expected, which could be inhibited by the NMDA receptor antagonist, AP-5. Similarly, addition of glutamate-induced increased spike and burst frequency, which could be inhibited by AP-5, indicating that glutamate acts via the NMDA receptor Fig. 4f (i) and (ii)). By contrast, the antagonist for the AMPA receptor, CNQX, failed to prevent NMDA/glutamate-induced neuronal hyperexcitation. This was an exact reflection of the hyper-excitatory phenotype that was observed when DIV90 ND neurons on MEA were treated with DMD ACM (Fig. 4f (i) and (ii)).

Finally, to assess the astrocyte/ACM specificity of the observed perturbations in the PSC neuronal networks, we evaluated the synapse forming ability of both ND and DMD neurons upon treatment with either ND or DMD ACM. After 30 days of maturation (further to initial 30 days to differentiation/derivation), we observed synaptic density as being dependent on neuro-supportive factors from astrocytes (ACM): addition of ND ACM increases while DMD ACM (D1, D2) decreased synaptic density of both ND and DMD neurons defined as the number of mature (colocalizing synapsin and PSD-95 staining) synaptic sites regardless of neuronal genotype i.e. on both ND and DMD DIV60 neuronal network (Fig. 4g (i) and (ii)).

### Premature stop codon read-through mediated increase of dystrophin corrects astrocyte mediated neuronal dysfunction

In a final set of studies, we used the small molecules/compounds (Readthrough compounds, RTC), gentamicin, G418 and PTC124 (Ataluren®), that were shown to allow read-through of premature termination codons (PTCs) in mutant genes (including in cystic fibrosis^44,63^ and DMD^56^), and hence re-establish protein levels of the gene products cystic fibrosis transmembrane conductance regulator (CFTR)^64^ or dystrophin, respectively, near levels in non-mutant cells. Preliminary experiments using high-throughput screening (HTS) in 96-well plates on an Operetta imaging system were performed to test RTC-mediated cytotoxicity (calcein/ethidium bromide staining) (not shown) and assess the RTC dose-responsive effect on astrocyte morphometry. We demonstrated a correction (mutant cell morphology/area reduced to normal range) only in the DMD line, DMD D2, with a premature termination codon mutation, in response to gentamicin (100 μg/mL or higher). Most importantly, this was associated with a significant increase in significantly increased dystrophin expression (Fig. S6A, B). Based on these results, and as PTC124 is used in DMD clinical trials and can cross the blood brain barrier (BBB) we selected to proceed with PTC124 as the ideal RTC, for rest of the experiments.

**Fig. 5:**
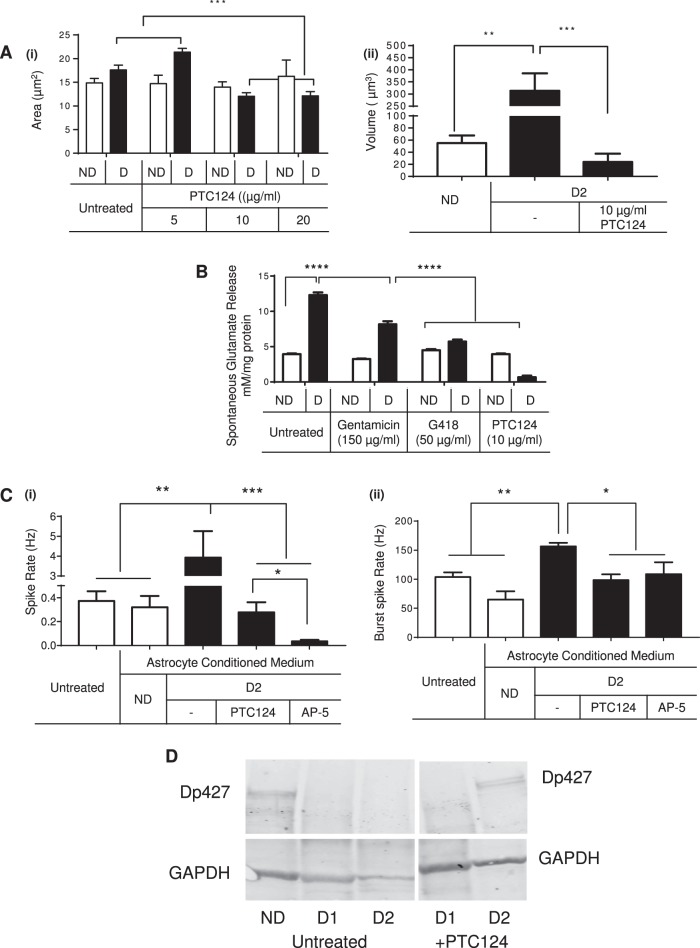
Rescue effects of dystrophin restoration in DMD astrocytes. **a** Morphometric parameters to describe the cytoskeletal organization in ND and DMD DIV120 astrocyte progeny with and without treatment with PTC124 (5, 10, and 20 µg/ml) for 7 days. Area (i), volume (ii). **b** Quantification of glutamate in medium conditioned by DIV120 ND (ChiPSC6B) and DMD-D2 astrocytes, harvested from cultures treated for 7 days with or without Gentamicin (150 µg/ml), G418 (50 µg/ml) and PTC124 (10 µg/ml). Data from ‘untreated’ are the same as in Fig. 2. **c** Multielectrode array (MEA) Recordings of ND (ChiPSC6B) DIV 90 neurons treated with Brainphys^67^ medium, with 60 min of ND (ChiPSC6B) DIV120 ACM, or 60 min with DIV120 ACM harvested from DMD-D2 cultures treated for 7 days with 10 mg/mL PTC124 or 30 min with the NMDA antagonist AP-5. (i) Spike rate; (ii) Burst spike rate. Data from ‘ND’ and ‘D2 untreated’ are the same as in Fig. 5. **d** Western blot of dystrophin isoform Dp427 in DIV120 ND (ChiPSC6B) astrocytes and DIV120 D1 and D2 astrocytes untreated or D1 and D2 treated for 7 days with 10 mg/mL PTC124, GAPDH was included as the loading control (samples not treated with PTC124 are similar as in Fig. 1i (ii)). All quantitative data is shown as Mean ± SEM of *n* > 10 technical replicates of each ND (ChiPSC6B) and DMD (D1, D2) cell lines. Significance was defined as *P < 0.01, **P < 0.001; ***P < 0.0001, ****P < 0.00001 based on one-way ANOVA analysis and Dunnett’s multiple comparison post-test against ND (+DMSO) controls. (Scale bar, 100 μm). Abbreviations: ND—Healthy control ACM, D—DMD ACM. In lieu of RTC treatment, all ND ACM samples were treated with DMSO

To further characterize the effectiveness of PTC124 to rescue dystrophic phenotypes in DMD PSC astroglial progeny, we first added three different concentrations of PTC124 to both DIV120 ND and DMD astrocytes and assessed cell area using ImageJ. PTC124, at 10 µg/ml, effectively reduced the hypertrophic nature of DMD-D2 astrocytes (Fig. 5a (i)), while the morphometry of ND astrocytes was unaffected. Also, 3D reconstruction analysis showed that the overall cell volume of DMD-D2 astrocytes became within normal range (Fig. 5a (ii)), upon PTC124 treatment.

Moreover, when we re-tested the concentration of glutamate in ACM from DIV120 DMD-D2 astrocytes treated with gentamicin, G418 and PTC124 (Fig. 5b), a significant decrease in glutamate was measured similar to those in ND ACM (ChiPSC6B and BJ1), with the most pronounced effects noted for PTC124.

We next applied medium pre-conditioned on either ND, D2 or PTC-treated DMD-D2 Astrocytes for 24 h (Fig. 5c (i) and (ii)). Compared to basal levels of spontaneous spike rate and burst spike rate i.e. under brainphys condition^65^, acute application of DMD-D2 ACM lead to an instantaneous increase in spike rate (and Burst spike rate). Not surprisingly, given that the excess glutamate in DMD-D2 ACM is likely the cause of induced hyperactivity, supplementation with AP-5 decreased the spike/burst rate down to levels comparable to Brainphys and ND ACM. Most importantly, treatment with PTC124 reversed the hyperactive spike/bust response. In addition, PTC treatment also restored altered gene expression of *GFAP*, *GLAST*, and *GLT-1* (Fig. S7).

To further demonstrate that the observed rescue with PTC124 was due to its RTC nature, i.e. restoration of dystrophin protein levels in otherwise dystrophin-deficient DMD-D2 astrocytes, we performed western blotting. Consistent with the initial immunostaining (Fig. 6SA, B), treatment with PTC124 restored expression of the major dystrophin isoform (Dp427) in DMD-D2 astrocytes (harboring a stop-codon point mutation in exon 36), where it was absent before treatment. By contrast, as expected, Dp427 was not detected in DMD-D1 astrocytes (harboring a large deletion of exons 49-52) following treatment with PTC124 relative to untreated cells (Fig. 5d).

These studies demonstrated thus that read-through compound-mediated restoration of dystrophin levels greatly reversed the defective astrocyte phenotype. We observed reversal to normal astrocyte morphology, we also observed a normalization of the abnormal glutamate handling and the subsequent hyperexcitability induced by DMD-D2 ACM on neuronal networks.

## Discussion

Formation of glutamate requires astrocytic pyruvate carboxylase (PC) and astrocytic tricarboxylic acid (TCA) cycle activity. Return of glutamate to neurons and continued release of glutamate depend on astrocyte glutamine synthetase (GS/*GLUL*) activity^16,17,32,33,35,36^. The dynamic metabolic fluxes in astrocytes and cellular localization of metabolic processes determine metabolic formation and degradation of glutamate. This study, for the first time, demonstrates that loss of dystrophin in DMD astrocytes causes defects in astrocyte glutamate handling (glutamate–glutamine shuttle), likely due to abnormal functionality of either *GLUL* and/or *EAAT* glutamate transporters (Fig. 3SC(ii)). The increased glutamate levels in ACM conditioned medium caused compromised dendritic growth and this resultant neuronal defect was reversed when neuronal glutamate receptors were blocked by antagonists. Importantly, defects in glutamate handling and subsequent neuronal defects were also reversed when dystrophin levels were restored using stop codon read-through drugs in the astrocytes from subject D2, definitively demonstrating a role of defective dystrophin levels in aberrant glutamate mediated neuronal defects. These new pathogenic insights in how neuronal functions can be affected by loss of astrocytic dystrophin in DMD patients open potential perspectives for management of the neurocognitive defects seen in DMD subjects.

In DMD, ±30 % of patients have different forms of neurocognitive defects, ranging from decreased IQ to ASD, ADHD, and OCD like phenotypes^2–4^. However, the mechanisms underlying these defects are ill understood. Neurocognitive defects can be caused by a number of genetic mutations, including mutations that affect the cell cytoskeleton, interfering with neurite outgrowth, sprouting and synapse formation of neurons^66,67^. In addition, evidence for astrocyte dysfunction/activation has also come from a post-mortem brain investigation in autism demonstrating increased mRNA levels of GFAP and observations of astrogliosis in the cerebellar white matter and subcortical white matter within the frontal lobe^21,68–70^.

Exploiting iPSC technology, we created cortical neuron and astrocyte progeny from ND and DMD PSCs. DMD cortical neuronal progeny did not display obvious intrinsic functional differences compared to ND cortical neuronal progeny (Fig. S1), including electrophysiological features on DIV90, assessed by MEAs. This is consistent with the fact that neurons do not express the major dystrophin isoform^7^ (Fig. S1). Although astrocyte generation was not impaired, we found significant morphometric abnormalities in DMD astrocytes, which is in line with defects seen in myofibers lacking dystrophin^62,71^(Fig. 2). In addition, we found that DMD iPSC-derived astrocytes displayed other defects similar to those found in dystrophic myofibers, including defects in Ca^+2^ homeostasis, NO signaling, ROS accumulation and increased mitochondria-specific ROS accumulation in response to mitochondrial toxicants^55^(Fig. 2). In addition to these ‘DMD myofiber-like’ phenotypes, we discovered that DMD iPSC-derived astrocytes also displayed significant defects in glutamate handling, and that the decreased glutamate uptake/consumption and increased *de novo* production by astrocytes caused neuronal defects, including aberrant neurite outgrowth and hyperexcitability. It is well known that extracellular glutamate controls several important neurological processes, including neuronal and glial cell differentiation and migration during development^72^. The concentration of glutamate in the extra-synaptic space is controlled by glutamate transporters, such as EAAT1 and EAAT2 on astrocytes^62,73,74^.

An association between the loss of the either Dp427 and/or Dp140 dystrophin protein isoform in DMD subjects and cognitive impairment has been suggested^75,76^. In addition, in DMD patients, altered glutamate levels have been identified in the brain^77^, as well as neuronal hyperexcitability. However, a direct causal link between loss of dystrophin protein and aberrant glutamate levels and subsequent neuronal defects has not been demonstrated^78^. Recent studies demonstrated in other disorders with a high incidence of ASD, such as fragile X syndrome or Rett syndrome^79–81^, that abnormal glutamate handling may at least in part underlie the observed neuropsychiatric defects. Likewise, in the absence of dystrophin, we observed a pathological profile that is characterized by both loss of glutamate homeostatic function and shades of reactive astrogliosis^32,33^. Dystrophin-deficient astrocytes in vitro seem to undergo remodeling where cell hypertrophy and downregulated expression of inward rectifying Kir4.1 channels is prominently observed. Furthermore, we detected up-regulation of intermediate filaments such as GFAP and vimentin, key features of reactive astrogliosis. We here demonstrate that DMD astrocytes released sufficient amounts of glutamate to induce widespread toxicity in cultured ND PSC-derived neuronal progeny. At the molecular level, sufficient DMD cell-released glutamate was present to activate NMDA and AMPA/KA receptors on cortical neurons, as an aberrant dendritic tree formation was evident when ACM was applied for 12 h on ND cortical neural progeny. The cause of the impaired glutamate handling by DMD cells, both removal/consumption and production, remains, however, to be determined. In addition, excess glutamate present in DMD ACM caused decreased neurite outgrowth by ND cortical neurons, as well as neuronal hyperexcitability. We demonstrated that this is directly correlated with absence of intact dystrophin, as glutamate handling was restored to ND-PSC astrocyte levels in DMD-D2 astrocytes, following treatment with the read-through molecule PTC124^53,82^. Moreover, our observation that ASD genes and pathways are enriched in the DMD-astrocyte transcriptome and that treatment with glutamate-rich DMD ACM induces increased neuronal network hyperactivity and abnormal neuronal morphology, lead us to speculate that increased susceptibility of the developing neuronal circuitry in DMD, might be caused by aberrant DMD astrocyte signaling leading to dysregulated synaptic plasticity^20,83–86^.

Although in the current study we demonstrate a causal link between absence of Dp427 and aberrant glutamate production and subsequent neuronal defects, we have not proven that there is a direct interaction between dystrophin and glutamate channels. Nevertheless, there is evidence that dystrophin associates with multiple channels and transporters in the cell membrane through syntrophin, for instance for AQP4 and Kir4.1^49,87^. Membrane proteins such as AQP4, harbor a PDZ binding motif which associates with the PDZ domain on the syntrophin peptides, allowing for proper localization into the plasma membrane^10^. In line with this, the glutamate channels, EAAT1 and EAAT2, also contain a PDZ binding domain^88–90^ (Fig. S8). Hence, we hypothesize that loss of dystrophin, and therefore the dystrophin scaffolding complex, may deregulate not only the function of Kir4.1 (also shown here to be reduced on the cell membrane of astroglial progeny) and AQP4, but also glutamate channels.

In DMD, most pharmacological treatments available or under investigation, focus on the skeletal/ respiratory/cardiac muscle component of the disease. However, little to no attention has been paid to neurocognitive or behavioral symptoms. Our finding that glutamate channel inhibitors or NMDA receptor blockers such as Memantine, could selectively inhibit the toxic effects of DMD ACM on neuronal morphology and function (neurite outgrowth and excitability) suggests that these inhibitors might hold exciting translational prospect to tackle the neurocognitive defects seen in DMD and other subjects. In addition, for the subpopulation of subjects with stop codon causing point mutations currently treated with PTC124, it will be of great interest to determine if this therapy also influences neurocognitive function.

## Supplementary information

Supplementary tables

Supplementary Figures 1-8

Supplementary_Legends_Methods
