# Supplementary material for: Dystrophin deficiency leads to dysfunctional glutamate clearance in iPSC derived astrocytes
Source: Transl Psychiatry. 2019 Aug 21;9:200. doi: 10.1038/s41398-019-0535-1 (PMC6704264; doi:10.1038/s41398-019-0535-1)
Supplement: Supplementary file 1 — Supplementary tables [file 41398_2019_535_MOESM1_ESM.pdf]

Supplementary Table 1: Patient Characteristics

ID	Cardiomyopathy	Behavioural Problems	ADHD	Autism
D1	No	No	NA	Yes
D2	Yes	No	NA	NA
D3	Yes	No	No	No
D4	No	No	No	No
D5	NA	Yes	NA	NA
D6	Yes	No	No	Yes

NA - Data Not Available

Supplementary Table 2: iPSC lines used

<table>
<tr><th>ID</th><th>Origin</th><th>Mutation</th><th>Category</th><th>iPSC Lines derived</th><th>Isoform Affected* (Expected)</th></tr>
<tr><td>D1</td><td>PBMC</td><td>Del. 49-52</td><td rowspan="6">DMD</td><td>2</td><td>Dp427, Dp260, Dp140</td></tr>
<tr><td>D2</td><td>Fibroblast</td><td>Pt.M 36 c.4996C>T(p.ARG1666X)</td><td>3</td><td>Dp427, Dp260</td></tr>
<tr><td>D3</td><td>PBMC</td><td>Del. 16-17</td><td>2</td><td>Dp427</td></tr>
<tr><td>D4</td><td>PBMC</td><td>Del. 51-55</td><td>2</td><td>Dp427, Dp260, Dp140</td></tr>
<tr><td>D5</td><td>PBMC</td><td>Del. 49-52</td><td>2</td><td>Dp427, Dp260, Dp140</td></tr>
<tr><td>D6</td><td>PBMC</td><td>Del. 46-51</td><td>3</td><td>Dp427, Dp260, Dp140</td></tr>
<tr><td>KSF-16-025#</td><td>Fibroblast</td><td>ND</td><td rowspan="5">Normal Donor (ND)</td><td rowspan="3"></td><td rowspan="3"></td></tr>
<tr><td>BJ1-iPSCs##</td><td>Fibroblast</td><td>ND</td></tr>
<tr><td>Sigma-iPSC0028</td><td>Fibroblast</td><td>ND</td></tr>
<tr><td>Cellectis – ChiPSC6B</td><td>Fibroblast</td><td>ND</td><td></td><td></td></tr>
<tr><td>H9 - ESCs</td><td>Blastocyst</td><td>ND</td><td></td><td></td></tr>
</table>

* DMD mutations database UMD-DMD France http://www.umd.be/DMD/W_DMD/index.html
The KSF-16-025 line is the SBAD2 line, obtained via P Jennings from STEMBANCC
Creation line described in Raitano *et al.,* Stem Cell Reports, 2005

Supplementary Table 3: Antibodies Used

Antigen	Dilution	Source	Identifier	
TUJI (beta 3 tubulin)	1/1000	Rabbit polyclonal	Synaptic systems	302302
SOX2	1/200	Rabbit IgG	Abcam	Ab5603
NESTIN	1/200	Mouse IgG1	Covance	656802
GFAP	1/200	Rabbit polyclonal	Dako Cytomation	334
Hoechst 33258	1/10000		Sigma	
PAX6	1/200	Mouse	Abcam	ab78545
FoxG1	1/300	Rabbit polyclonal	Abcam	ab 18259
MAP2	1/300	Guinea pig polyclonal	Synaptic systems	188002
anti-Doublecortin	1/400	Guinea Pig	Millipore	AB 2253
Tbr1	1/500	Rabbit IgG	Abcam	ab31940
AQP4	1/400	Rat	Alomone	AQP-014
EAAT1	1/500	Rabbit Polyclonal	Alomone	AGC-021
EAAT2	1/200	Rabbit Polyclonal	Alomone	AGC-022
Phalloidin	1/200		ThermoFisher Scientific	
Vimentin	1/300	Mouse monoclonal	Dako Cytomation	
OCT-3/4	1/300	Mouse monoclonal	Santa Cruz	sc-5279
TRA-1-60	1/250	Mouse monoclonal	Millipore	MAB4360
S100β	1/1000	Mouse	Abcam	ab11178
DYSTROPHIN	1/2000	Rabbit	Abcam	ab154168
β sarcoglycans	1/200		Novocastra	NCL-L-b-SARC
GAPDH	1/10000	Rabbit	Abcam	128915

Type	Fluorochrome	Dilution	Provider
Goat anti mouse IgM	AF-555	1/500	Life technologies
Rabbit anti mouse IgM	FITC	1/500	Life technologies
Donkey anti-Goat IgG	AF-555	1/500	Life technologies
Donkey anti-Goat IgG	AF-488	1/500	Life technologies
Goat anti chicken IgY	AF-488	1/500	Life technologies
Goat anti chicken IgY	AF-555	1/500	Life technologies
Donkey anti chicken IgY	AF-488	1/200	Jackson laboratories
Goat anti mouse IgG	AF-555	1/500	Life technologies
Goat anti Rabbit IgG	AF-488	1/500	Life technologies
Goat anti Rabbit IgG	AF-555	1/500	Life technologies
Donkey anti Rabbit IgG	AF-647	1/500	Life technologies
Donkey anti Goat IgG	AF-555	1/500	Life technologies
Donkey anti Goat IgG	AF-488	1/500	Life technologies
Donkey anti Rabbit IgG	AF-488	1/500	Life technologies
Donkey anti Rabbit IgG	AF-555	1/500	Life technologies
Donkey anti Rat IgG	AF-488	1/500	Life technologies
Donkey anti Chicken IgY	Cy3	1/500	Life technologies

Supplementary Table 4: Human qPCR Primers

Gene	Forward Primer	Reverse Primer
GAPDH	tcaagaaggtggtgaagcagg	accaggaaatgagcttgacaaa
OCT4	GATGGCGTACTGTGGGCCC	TGGGACTCCTCCGGGTTTTG
Nestin	TCAGCTTTCAGGACCCCAAG	TGGGAGCAAAGATCCAAGACG
Pax6	AGGCCCTGGAGAAAGAGTTTG	TTTGGCTGCTAGTCTTTCTCG
FoxG1	CGTCCACCATATAGTTCCATGA	TGACTGCTTTGCCATTTCATTC
Sox2	GAGTGGAAACTTTTGTCGGAGA	AGCGTGTACTTATCCTTCTTCAT
Sox1	CCACATCCTAATCTTGAGCCA	CTGACGTCCACTCTCAGTCT
Tbr2/EOMES	GCTCAAGAAAGGAAACATGCG	CACGTCTACCTGTGCAACC
Otx2	CATTCTGCTGTTGTTGCTGTT	GCTGAGTCTGACCACTTCG
Tbr1	CGTGTCATAATTATCCCGAAATCC	CAGACGTTCACTTTCCCTGAG
CTIP2/BCL11B	GTTGTGCAAATGTAGCTGGAA	GAAGATGACCACCTGCTCTC
vGlut1/SLC17A7	ACTGGCATAGACGTGAAGAAG	GGAGCGCAAGTACATCGAG
BLBP/FABP7	GGATAGCACTGAGACTTGAGG	AGAGAAATTAAGGATGGCAAAATGG
BIII tubulin/TUBB3	CCTCCGTGTAGTGACCCTT	GGCCTTTGGACATCTCTTCAG
GFAP	GAGATCCGCACGCAGTATGA	TCTGCAAACTTGGAGCGGTA
S100B	CACATTCGCCGTCTCCATC	CACAAGCTGAAGAAATCCGAAC
CD44	GAGATGCTGTAGCGACCATT	GACACCATGGACAAGTTTTGG
AQP4	TGGACAGAAGACATACTCATAAAGG	GGTGCCAGCATGAATCCC
ALDH1L1	CTTCCAGGACAGCATCATCAG	CACCATCCCCATCAACCAG
GLT1/EAAT2	ATCTTGGCTCAGAGGAACCCA	CAGGATGACACCAAACACCG
GLAST/EAAT1/SLC1A3	TAATAGACTACAGCTCGCATTCC	GAGGATGTTACAGATGCTGGTC
ApoE	GTTGTTCCTCCAGTTCCGATT	TCTGAGCAGGTGCAGGA
SPARCL1	GAGGATGCTGGAAAGTTGAGT	CCGTTTCTTTGAGGAGTGTGA
KCNJ1	GCACGTTCTAATACAGTAGCCT	GCCTTCAAAGTCTACCAGCA
All Dystrophin isoforms	Retrieved from Leiden Muscular Dystrophy pages© Center for Human and Clinical Genetics, Leiden University Medical Center	http://www.dmd.nl/

Supplementary: Key Resources Table

REAGENT or RESOURCE	SOURCE	IDENTIFIER
Critical Commercial Assays		
60-mer oligonucleotide microarrays hybridization	Agilent	
Cytotune 2	ThermoFisher Scientific	A16517
Cytotune 1	ThermoFisher Scientific	(A1378001)
DNeasy	(Qiagen),	
GenElute™ Mammalian Total RNA Miniprep Kit		
Glutamate Fluorometric Assay Kit	Abcam	
Glutamate Glo		
LightCycler 480 SYBR Green I Master Mix	Roche	4707516001
microBCA kit	ThermoFisher Scientific	
MicroPrep	Zymo Research, CA, USA	
RIPA buffer		
RNA isolation RNeasy Mini Kit	QIAGEN	74106
SuperScript® III First-Strand Synthesis		
SYBR Green PCR Master Mix	Applied Biosystems	4309155
TaqMan® hPSC Scorecard™ Panel (Life Technologies—A15870—HPS scorecard panel 384)		
TaqMan® SNP Genotyping Assay kit		
Software and Algorithms		
- CIBERSORT		
Agilent Feature Extraction software	Applied Biosystems	https://www.thermofisher.com/de/de/home/technical-resources/software-downloads/applied-biosystems-viia-7-real-time-pcr-system.html
Allen Brain Atlas		
AxioVision V4.6.3.0 software		
Database for Annotation, Visualization and Integrated Discovery (DAVID)	GraphPad Software, Inc.	http://www.graphpad.com
FACS DIVA software (Becton & Dickinson).		https://david.ncifcrf.gov/
Flow Jo (FlowJo, LLC, USA)		http://bioconductor.org/packages/release/bioc/html/lumi.html
Gene set enrichment analysis (GSEA, v2.2.1)		http://human.brain-map.org/static/download
GraphPad Prism Windows 5.04	Subramanian et al., 2005	http://software.broadinstitute.org/gsea/index.jsp
IPA (Qiagen)		
Neurite Tracer within the FIJI software package	ImageJ	
Neuronstudio		
Operetta: with PhenoLOGIC (PerkinElmer)		
R/Bioconductor packages in the programming language R (version 3.1 or greater)		
Simple Neurite Tracer, and L-Measure6	ImageJ	
ViiA 7 Software		
Hardware		
AxioCam MRc5 (bright field)	Carl Zeiss	
AxioCam Mrm camera (fluorescence)	Carl Zeiss	
Axioimager.Z1 microscope		
Hamamatsu ORCA-Flash high speed camera	Hamamatsu Photonics	
Nikon C2 Eclise Ni-E Confocal confocal microscope	Nikon, Tokyo, Japan)	
humidified incubator (5% CO2, 37°C)		
multiwell multielectrodearray (MEA) system		
Nanodrop ND-1000 spectrophotometer ()	Nanodrop Technologies	
Olympus IX71 widefield microscope system	Olympus	
Operetta High Content Screening (HCS) System	PerkinElmers	
ViiA™ 7 Real-Time PCR System	Applied Biosystems, Carlsbad, USA	
Consumables		
CellCarrier 96 well plate	PerkinElmer	
18 mm glass coverslips	VWR	, 831-0153
microwell dishes	MatTek	
6-well plates	Corning	

Supplementary Table 1: Patient Characteristics

ID	Cardiomyopathy	Behavioural Problems	ADHD	Autism
D1	No	No	NA	Yes
D2	Yes	No	NA	NA
D3	Yes	No	No	No
D4	No	No	No	No
D5	NA	Yes	NA	NA
D6	Yes	No	No	Yes

NA - Data Not Available

Supplementary Table 2: iPSC lines used

<table>
<tr><th>ID</th><th>Origin</th><th>Mutation</th><th>Category</th><th>iPSC Lines derived</th><th>Isoform Affected* (Expected)</th></tr>
<tr><td>D1</td><td>PBMC</td><td>Del. 49-52</td><td rowspan="6">DMD</td><td>2</td><td>Dp427, Dp260, Dp140</td></tr>
<tr><td>D2</td><td>Fibroblast</td><td>Pt.M 36
c.4996C>T(p.ARG1666X)</td><td>3</td><td>Dp427, Dp260</td></tr>
<tr><td>D3</td><td>PBMC</td><td>Del. 16-17</td><td>2</td><td>Dp427</td></tr>
<tr><td>D4</td><td>PBMC</td><td>Del. 51-55</td><td>2</td><td>Dp427, Dp260, Dp140</td></tr>
<tr><td>D5</td><td>PBMC</td><td>Del. 49-52</td><td>2</td><td>Dp427, Dp260, Dp140</td></tr>
<tr><td>D6</td><td>PBMC</td><td>Del. 46-51</td><td>3</td><td>Dp427, Dp260, Dp140</td></tr>
<tr><td>KSF-16-025#</td><td>Fibroblast</td><td>ND</td><td rowspan="5">Normal Donor (ND)</td><td rowspan="3"></td><td rowspan="3"></td></tr>
<tr><td>BJ1-iPSCs##</td><td>Fibroblast</td><td>ND</td></tr>
<tr><td>Sigma-iPSC0028</td><td>Fibroblast</td><td>ND</td></tr>
<tr><td>Cellectis – ChiPSC6B</td><td>Fibroblast</td><td>ND</td><td></td><td></td></tr>
<tr><td>H9 - ESCs</td><td>Blastocyst</td><td>ND</td><td></td><td></td></tr>
</table>

* DMD mutations database UMD-DMD France http://www.umd.be/DMD/W_DMD/index.html
The KSF-16-025 line is the SBAD2 line, obtained via P Jennings from STEMBANCC
Creation line described in Raitano *et al.,* Stem Cell Reports, 2005
