# Supplementary material for: Dystrophin deficiency leads to dysfunctional glutamate clearance in iPSC derived astrocytes
Source: Transl Psychiatry. 2019 Aug 21;9:200. doi: 10.1038/s41398-019-0535-1 (PMC6704264; doi:10.1038/s41398-019-0535-1)
Supplement: Supplementary file 3 — Supplementary_Legends_Methods [file 41398_2019_535_MOESM3_ESM.pdf]

Supplementary Materials

Supplementary Figure 1: ND and DMD iPSCs derivation and characterization

Supplementary Figure 2. Cortical neurogenic differentiation from DMD iPSC

Supplementary Figure 3: Electrophysiology of PSC-Astrocytes and Calcium Transients

Supplementary Figure 4: Modular and Gene set overlap analysis of RNAseq Data

Supplementary Figure 5: Phenocopy DMD ACM phenotype by exogenous glutamate to ND neurons

Supplementary Figure 6: Highthroughput screen (HTS) to evaluate Dystrophin restoring ability of known readthrough aminoglycoside, Gentamicin

Supplementary Figure 7: qPCR gene expression changes upon PTC treatment

Supplementary Figure 8: Proposed hypothetical model of EAAT dysfunction in dystrophin deficient Astrocytes

Supplementary Table 1: Subject Characteristics

Supplementary Table 2: iPSC lines used

Supplementary Table 3: Antibodies Used

Supplementary Table 4: Human qPCR Primers

Supplementary: Key Resources Table

Supplementary Methods

Supplementary Figure 1: ND and DMD PSCs derivation and characterization

(A) FACS analysis of pluripotency markers in ND (ChiPSC6B line) and DMD lines (D3, D4) (TRA1-60-Top) and (SSEA4 – Bottom). Representative plot for each iPSC line.

(B) Immunostaining of pluripotency-associated markers for the DMD D3 iPSC lines. Upper row, co-expression of pluripotent stem cell markers NANOG (in green) and Tra1-81 (in red, cell surface localization). Middle row, co-expression of pluripotent stem cell markers OCT4 (in green) and TRA-1-60 (in red). Lower row, co-expression of pluripotent stem cell markers SOX2 (in green) and SSEA4 (in red). Similar results were found for all iPSC lines generated. (Scale bar, 100 µm). Representative images for each iPSC line.

(C) Representative histologic sections of a teratoma (two teratomas were generated for each iPSC line) from an immunodeficient mouse injected with DMD-D2 iPSC. Top to bottom: Neuroepithelium, Hyaline cartilage and Gut endothelium.

(D) EB based differentiation assay and subsequent qPCR analysis using the scorecard algorithm for the iPSC line DMD (D3, D4 and D6). Similar results were found for all iPSC lines generated. Representative qRT-PCR validation of pluripotency and the elimination of Sendai Viral vectors in the DMD-D2 iPSC line. Similar results were found for all iPSC lines generated.

Supplementary Figure 2. Cortical neurogenic differentiation from DMD iPSC

(A) Schematic of Neural Stem Cell (NSC) generation from hPSCs by dual SMAD inhibition

(B) Representative Immunostaining for NESTIN (red) and PAX6 (green) and Hoechst for nuclear staining (blue) of NSCs from ND (BJ1-iPSC (i)) and DMD (D1 (ii)) iPSC on DIV14. Scale bar represents 100 µm. Quantification of number BLBP (III) and FOXG1 (IV) expressing NSCs by immunostaining of DIV14 progeny. Transcript levels of *OTX2* (v) and *FOXG1* (VI) in NSCs from ND and DMD PSC, on DIV14. Levels shown as CT value of gene of interest minus CT value for housekeeping gene GAPDH (delta-CT). Mean ± SEM of n ≥ 4 biological replicates of 2 ND (ChiPSC6B and BJ1-iPSCs) and 4 DMD (D1, D2, D3, D5) lines.

(C) Representative Immunostaining of ND (BJ1-iPSC (I)) and DMD (D2 (ii)) neurons with an antibody against B3T (red), the deep layer identity marker TBR1 (green) and Hoechst for nuclear staining (blue) at DIV30. Scale bar represents 100 µM. Quantification of number TBR1(iii) expressing NSCs ND (ChiPSC6B) and DMD (D1, D2, D3, D5) -derived neural progeny. Transcript levels of the deep layer markers *TBR1* (iv); *CTIP2* (v) and glutamatergic synapse *vGLUT1* (vi) in DIV30 neural progeny from ND and DMD PSCs. Levels shown as CT value of gene of interest minus CT value for housekeeping gene *GAPDH* (delta-CT). (Mean ± SEM, n ≥ 4 biological replicates; 2 ND (ChiPSC6B and BJ1-iPSCs) and 4 DMD (D1, D2, D3, D5) lines.

(D) Electrophysiological recordings using multi-well multielectrode array (MEA), Spikes rate (i) and Burst spikes rate (ii) were recorded as indicated in the bar graphs. Mean ± SEM, n = 4 biological replicates, 2 ND (ChiPSC6B and Bj1-iPSCs) and 4 DMD (D1, D2, D3, D5) lines.

(E) qRT-PCR analysis for dystrophin isoforms expression in either 2 ND (ChiPSC6B, Bj1-iPSCs) or 4 DMD lines (D1, D2, D3, D5) cortical neurons at DIV30.

Supplementary Figure 3: Electrophysiology of PSC-Astrocytes and Calcium Transients

(A) Patch clamp studies on ND (ChiPSC6B line) and DMD (D1, D2) DIV120 astrocytes. Whole-cell capacitance of ND and DMD astrocytes (n = 30–35 cells, (i)). Resting membrane potential (Vm) (n = 30–35 cell, (ii)).

(B) Current traces from a representative ND (ChiPSC6B line) astrocyte (i) and a DMD (D2) astrocyte (ii) under voltage clamp at different voltages (400 ms, 10 mV steps from −80 to + 50 mV).

(C) Representative immunostaining analysis of characteristic astrocytic markers GLAST (red) (i) and Kir4.1 (red)(ii) (green) and Hoechst for nuclear staining (blue) of astrocyte progeny from ND (BJ1-iPSC, H9) and DMD (D1, D2) iPSC on DIV120. Scale bar represents 100 µm.

(D) Calcium transient triggered by application of 1 mM ATP stimuli in DIV120 ND (ChiPSC6B line) and DMD (D1, D2) astrocytes.

Supplementary Figure 4: Modular and Gene set overlap analysis of RNAseq Data

(A) Weighted correlation network analysis (WGCNA) Dendrogram showing clusters of highly correlated genes into modules based on topological overlap. Each colored domain i.e. Yellow, Blue, Brown and Turquoise, corresponds to a distinct module as determined by the dynamic tree cutting algorithm

(B) Two of the prominent WGCNA modules (Turquoise and Blue), were enriched with genes involved in synaptic activity and ECM, respectively.

(C) Enrichment analysis of genes associated with autism spectrum disorder (ASD) and other brain disorders in the ND and DMD differentially expressed gene transcriptome dataset. The x-axis indicates −log10 (B-H corrected P-value) i.e. significance level of gene list(s) overlap. All enrichment values for the gene set overlap analysis enriched at $P < 0.05$ (one-sided Mann-Whitney U-test; FDR-corrected) are shown (i). Also indicated are normalized read count value for *GLUL* transcript in ND and DMD astrocytes (ii).

(D) Indicates pathways that are highly enriched between DMD and ASD transcriptomes based on overlapped genes, as assessed via the IPA algorithm.

Supplementary Figure 5: Phenocopy DMD ACM phenotype by exogenous addition of glutamate to ND neurons

(A) Titration curve to determine toxic concentration of glutamate that would compromise the Euclidean distance in ND neurons. X-axis represents increasing Glutamate concentration in µM, while y-axis displays the Euclidean distance in µm. (shown is Healthy, ChiPSC6B line). Each dot represents a mean a technical replicate, from two independent experiments at each concentration

(B) Morphometric analysis of DIV30 ND neurons treated with either direct glutamate application alone (i) or in combination with antagonist of ionotropic glutamate receptors (AMPAR via CNQX and NMDA via AP-5) (ii). Mean ± SEM, n ≥ 4 biological replicates; 2 ND (ChiPSC6B, Bj1-iPSCs) and 4 DMD lines (D1, D2, D3, D5).

Supplementary Figure 6: High throughput screen (HTS) to evaluate Dystrophin restoring ability of known readthrough aminoglycoside, Gentamicin.

(A) Representative Immunostaining of ND (BJ1-iPSC) and DMD (D2) DIV120 Astrocytes stained with n antibody against Dystrophin (red), Phalloidin (F-Actin) and Hoechst for nuclear staining (blue); both untreated (i) and 150 µg/ml Gentamicin (ii). Scale bar represents 100 µM. Analysis was done on an Operetta HTS system.

(B) Dystrophin staining intensity quantified in 1 ND (BJ1-iPSC), and two DMD (D1, D2) DIV astrocyte upon Gentamicin treatment and normalized to β-dystroglycan staining intensity which is not affected in DMD lines. Significance was defined as *P < 0.05, **P < 0.01, ***P < 0.001 or ****P < 0.0001 based on one-way ANOVA analysis and Dunnett's multiple comparison post-test against 0 µg/ml treatment condition.

Supplementary Figure 7: qPCR gene expression changes upon PTC treatment

Transcript levels of a *GFAP*, *GLAST* and *GLT-1* in ND, DMD, and D2 DIV120 astrocytes. Levels shown as relative gene expression, CT value of gene of interest divided by CT value for housekeeping gene (GAPDH). n = 4 biological replicates, one column of 384 well qPCR plate (i.e. 16 wells) was used as technical replicates for each of n=4 runs. All analyses based on Student's t-test, * P< 0.01, **P<0.001

Supplementary Figure 8: Proposed hypothetical model of EAAT dysfunction in dystrophin deficient Astrocytes

Proposed model for the proper localization of glutamate transporter(s), also known as Excitatory Amino Acids Transporters (EAATs) to the astrocytic plasma membrane. EAATs likely interacts with Dystrophin protein via members of syntrophin family of adaptor proteins. Wherein, the PDZ binding motif of EAATs binds to PDZ domain in syntrophin, which in turn binds directly to multiple spectrin-like repeats in dystrophin.

Supplementary Table 1: Patient Characteristics

Summary of DMD subjects used in the current study and their physiological and psycho-cognitive anomalies, as reported by the clinicians, up to date as of Feb 2015.

Supplementary Table 2: PSC lines used

Summarized here are the origins of both normal donor and DMD pluripotent stem cell lines. For DMD lines, each line received an ID (D1, D2, D3, D4, D5 and D6). Also indicated are the mutation(s) harbored within the lines.

Supplementary Table 3: List of primary and secondary antibodies used

Supplementary Table 4: List of primers used

Key Resource Table

Supplementary Methods

Embryoid body, Teratoma Formation and Analysis

The embryoid body assays were performed as previously described and The TaqMan® hPSC Scorecard™ Panel (Life Technologies—A15870—HPS scorecard panel 384, Belgium) kit was used to determine the expression of markers from the three germ layers.

For teratoma formation, iPSCs were collected through enzymatic dissociation, 5–10 × 10^6 cells were resuspended in 100 µl phosphate-buffered saline (PBS, Gibco, USA) and injected with 100 µl Becton Dickinson matrigel (BD) subcutaneously under the skin of Rag2 *(-/-)* γc *(-/-)* mice, using a protocol approved by the Institutional Ethics Committee at KU Leuven. After four weeks, tumors were harvested, fixed, and sectioned to assess the presence of cells from the three germ layers following hematoxylin and eosin staining.

Genotype confirmation and quality control

Human single nucleotide polymorphisms (SNP) assays were performed on the original, starting cell population i.e. PBMCs and Fibroblasts, as well as on subsequently derived cell types including iPSC, Neural stem cells (NSCs) and Astrocytes. Briefly, Genomic DNA was isolated using DNeasy (Qiagen, Belgium), according to the manufacturer's instructions. DNA concentrations were measured on a Nanodrop ND-1000 spectrophotometer (Nanodrop Technologies, ThermoFisher Scientific). TaqMan® SNP Genotyping Assay kit (ThermoFisher Scientific) was used as per manufacturer's instructions to check the presence or absence of selected SNPs among all cell types.

Also, MLPA and Sanger sequencing were performed on PBMCs/Fibroblasts, iPSCs and DIV120 Astrocytes at the Center for Human Genetics (Centrum Menselijke Erfelijkheid, CME), in Leuven and at the Laboratory for Diagnostic Genome Analysis (LDGA) in Leiden to confirm the presence of the specific mutation. Lastly, comparative genomic hybridization (array-based CGH) was performed on all iPSCs generated. Genomic DNA isolation as described for SNP analysis, DNA copy number variations were detected via Agilent 60-mer oligonucleotide microarrays hybridization, as per manufacturer's instructions (Agilent Technologies, Belgium). Final readout was obtained using Agilent Feature Extraction software in Agilent CytoGenomics Workbench, and analyzed by a clinical geneticist, Dr. Kris Van Den Boogaert, UZ Gasthuisberg, Leuven.

Flow Cytometry

Cells were enzymatically harvested using accutase (ThermoFisher Scientific), centrifuged and resuspended in 100 µl FACS buffer (PBS 1x, 2% fetal bovine serum and 0.02% sodium azide).

For Surface antigen staining (SSEA4, TRA1-60, TRA1-81; all antibodies and isotype controls from BD Biosciences) manufacturer's instructions were followed.

Flow cytometry was performed at the KU Leuven Flow Cytometry Facility. All results were analyzed using Flow Jo (FlowJo, LLC, USA) and FACS DIVA software (BD). Gating was done using unstained cells and appropriate isotype control antibody stained samples.

Transcriptome analysis and RNA sequencing Deconvolution

Total RNA was isolated using the QIAGEN isolation kit (QIAGEN) and quality-checked by Nanodrop analysis (Nanodrop Technologies) from DIV120 astrocytes. RNA integrity was assessed using a Bioanalyzer 2100 (Agilent). Per sample, an amount of 100 ng of total RNA was used as input. Using the Illumina TruSeq® Stranded mRNA Sample Prep Kit (protocol 15031047 Rev.E "October 2013") poly-A containing mRNA molecules were purified from the total RNA input using poly-T oligo-attached magnetic beads. In a reverse transcription reaction using random primers, RNA was converted into first strand cDNA and subsequently converted into double-stranded cDNA in a second strand cDNA synthesis reaction using DNA Polymerase I and RNAse H. The cDNA fragments were extended with a single 'A' base to the 3' ends of the blunt-ended cDNA fragments after which multiple indexing adapters were ligated, introducing different barcodes for each sample. Finally, enrichment PCR was carried out to enrich DNA fragments that had adapter molecules on both ends and to amplify the DNA in the library. Sequence libraries of each sample were equimolar pooled and sequenced on an Illumina NextSeq 500 instrument (High Output, 75 bp, Single Reads, v2) at the VIB Nucleomics core (www.nucleomics.be). Subsequently, low-quality ends and adapter sequences were trimmed off from the Illumina reads. Subsequently, small reads (length < 35 bp), ambiguous reads (containing N), and low-quality reads (more than 50% of the bases < Q25) were filtered. Processed reads were aligned with Tophat, v2.0.8b, to the human reference genome (GRCh38), as downloaded from the Genome Reference Consortium (https://www.ncbi.nlm.nih.gov/grc/human/data). Default Tophat parameter settings were used, except for "min-intron-length = 50", "max-intron-length = 500,000", "no coverage-search" and "read-realign-edit-dist = 3".

3D Principal Component Analysis (PCA) was used to visualize multiple transcriptome correlations using the R/Bioconductor packages in the programming language R /version 3.1 or greater). Differentially

expressed genes (DEGs) were identified based fold change of two or more; comparing ND (H9-ESCs and ChiPSC6B –iPSCs derived), and DMD Mutations Astrocytes samples using the DESeq2 software package and adjusted p values (padj) <0.05). Each gene value was further assigned as their relative abundance value, which corresponded to the expression value of the gene in each sample divided by its mean expression value across the samples. Volcano plots, Gene Ontology (statistical significance ($P < 0.05$)) and weighted correlation network analysis (WGCNA) were derived and assembled in iDEP (integrated Differential Expression and Pathway analysis) web application. Ingenuity pathway analysis (IPA, Qiagen), was performed with build 456367, content version 39480507.

To assess the transcriptome of ND and DMD PSC-astrocytes with the published transcriptional signature of PSC derived neurons, astrocytes and oligodendrocytes, firstly, Individual data sets underwent stringent quality control, normalization and batch correction using ComBat function (from the sva package in R), followed by RNAseq deconvolution. This was performed using custom made signature files (Single cell data of transcripts most highly enriched in each of the neural cell types). Next, the signature and ND/DMD DEGs files were uploaded to the CIBERSORT web platform and the algorithm was run to compare transcript profiles of astrocytes analyzed herein. Output indicates mRNA fraction of transcripts defining a given cell identity.

Gene set enrichment between DMD DEGs and ASD

ASD candidate genes were downloaded from the SFARI database (https://gene.sfari.org/autdb/GS_Home.do) and the AutismKB. The two schizophrenia gene lists were from the SZgene database and a recent GWAS report. Bipolar disorder associated genes were from the BDgene database. For shared spatial expression analysis, we utilize FUMA, an integrative web-based platform using information from multiple biological resources such as 56,320 genes from GTEx, to facilitate functional annotation of GWAS results, gene prioritization and interactive visualization.
